# A defined microbiota mouse model for *Salmonella* Paratyphi A oral infection

**DOI:** 10.3389/fmicb.2026.1824783

**Published:** 2026-05-19

**Authors:** Caleb Skow, Logan C. Ott, Michael J. Wannemuehler, Melha Mellata

**Affiliations:** 1 Interdepartmental Graduate Microbiology Program, Iowa State University, Ames, IA, United States; 2 Department of Food Science and Human Nutrition, Iowa State University, Ames, IA, United States; 3 Veterinary Microbiology and Preventive Medicine, Iowa State University, Ames, IA, United States

**Keywords:** ASF mice, disease model, knockout mice, paratyphoid, typhoidal *Salmonellae*

## Abstract

**Introduction:**

*Salmonella* Paratyphi A (SPtA) is an emerging pathogen that primarily infects humans and causes enteric fever, yet it has not received sufficient research attention. To facilitate further investigation, an appropriate animal model is necessary for testing SPtA infection.

**Methods:**

In this study, we utilized gnotobiotic mice harboring the altered Schaedler flora (ASF) from either C3H/HeN or 129S6/SvEv genetic backgrounds. These mice, subjected to various inflammatory conditions, were orally challenged with SPtA. The colonization and persistence of the pathogen were assessed in both intestinal and extraintestinal tissues, while also examining changes in gut microbiota, the expression of tight junction proteins, cytokines, and histopathological changes.

**Results:**

Our findings revealed that both the inflammatory state and the sex of the ASF mice significantly affected the colonization of SPtA. Notably, infection of the 129S6/SvEv *IL10^−/−^* mice with SPtA replicated key features observed in humans. This included the extraintestinal dissemination of SPtA, the inhibition of pro-inflammatory cytokines, and progressively worsening pathological changes over time following infection in *IL10^−/−^* ASF mice.

**Conclusion:**

The results of this study suggest that ASF mice represent a suitable low-cost model for investigating the pathogenesis of SPtA infection. The presence of ASF creates an open niche for SPtA colonization, allowing for the retention of a defined microbiome. This enables researchers to study perturbations in the microbiome without the limitations inherent to an antibiotic-treated model alternative. Furthermore, the chronic inflammatory conditions associated with *IL10^−/−^* phenotype enable SPtA extraintestinal invasion and disease progress in the murine host, facilitating a functional model of SPtA pathogenesis.

## 1 Introduction

An estimated five million cases of paratyphoid fever occur worldwide yearly (Appiah et al., 2020). In developing countries, the occurrence of paratyphoid fever is particularly high among travelers (Crump and Mintz, 2010; Meltzer and Schwartz, 2010; Appiah et al., 2020; Piovani et al., 2024). *Salmonella* (*S.*) *enterica* Typhi (ST) and *S. enterica* Paratyphi A (SPtA) are both host-restricted pathogens that primarily infect humans and other primates closely related to humans, which, in conjunction with a concerningly low burden-adjusted research intensity, further necessitates the development of a suitable model for research (Gibani et al., 2018; Furuse, 2019; Appiah et al., 2020). However, using humans as models for infections is ethically and financially challenging. Both ST and SPtA are causative agents of paratyphoid fever, which is endemic to Africa, Latin America, and Asia, although ST is vaccine-preventable (Appiah et al., 2020; Patel et al., 2024). The mortality rate associated with SPtA infection is increasing due to the absence of a vaccine for the prevention of paratyphoid fever, and neither of the two currently licensed typhoid fever vaccines, Ty21a (Vivotif, Emergent BioSolutions) and ViCPS (Typhim Vi, Sanofi Pasteur), licensed by the United States Food and Drug Administration (FDA), prevents paratyphoid fever (Jackson et al., 2015; Appiah et al., 2020).

To expedite the timeline of the SPtA research and develop an acceptable treatment for human diseases, it is necessary to use a non-human model for SPtA infection. Initial studies should focus on treatments targeting SPtA (Kiros et al., 2012). Historically, research on typhoidal *Salmonella* has utilized *S. enterica* Typhimurium (STm), an *S. enterica subsp. enterica* serovar that causes typhoid-like disease in mice (Hormaeche, 1979b; Barthel et al., 2003; Hapfelmeier and Hardt, 2005; Chaudhuri et al., 2018; Schultz et al., 2018; Nilsson et al., 2019). However, a disease-specific model is much more attractive as many of the virulence effectors, such as the *Salmonella* protein tyrosine phosphatase and Gifsy-2 bacteriophage-encoded effector GtgE (Ho et al., 2002) integral to STm pathogenesis, are either absent or present only as pseudogenes in STm and SPtA (Gal-Mor et al., 2014; Johnson et al., 2018). Due to SPtA’s nature as a human, host-adapted pathogen, identifying an acceptable animal model for disease has been difficult (Levine et al., 2011).

Conventional mice infected with non-Typhimurium *Salmonella* typically show no clinical signs of disease, such as fever or death, which are characteristic of Typhoidal *Salmonella,* except in cases of peritoneal injection (Hormaeche, 1979b, 1979a; Healing, 1991; National Research Council, 1991; Meerburg and Kijlstra, 2007). The extraintestinal invasion of SPtA varies by strain and transmission, and infection is not replicated in mice as it is in humans (i.e., fecal–oral). Adverse health effects are generally difficult to differentiate between true septicemia and endotoxemia (Carter and Collins, 1974; Pasetti et al., 2003). Research has demonstrated that mice pre-treated with streptomycin (i.e., depletion of resident microbiota) and subsequently given oral inoculations of SPtA can experience intestinal colonization and invasion of spleen and lymph nodes. However, there are several flaws in this model, including inconsistencies in microbiome composition that impact repeatability, the selection for resistant microbes that may arise, and potential negative impacts on the host cells (Elhadad et al., 2016; Kennedy et al., 2018).

The use of germ-free models ensures sterility pre-inoculation and lacks the flaws of the antibiotic-treated model (Kennedy et al., 2018). However, the lack of a microbiota leads to immune developmental deficits, such as the inability to effectively produce an antibody response to immunization or activated macrophages. These deficits can drastically diminish the translatability of results to human disease caused by SPtA (Lamousé-Smith et al., 2011; Kennedy et al., 2018). Models for the alternate human-restricted typhoidal *Salmonella*, ST, have been generated through genetic manipulation of key innate immune receptors, as is the case with *tlr1*^*1*−/−^ mice, and humanization of genetically manipulated models, such as hu-SRC-SCID grafted NOD-SCID *IL2ry^null^* mice (Libby et al., 2010; Mathur et al., 2012).

Neonatal mice have historically been employed as a model for studying oral infections associated with host-restricted pathogens, such as *Vibrio cholerae*. They have also been employed to study the pathogenesis of typhoidal *Salmonellae* (Chaicumpa and Rowley, 1972; Javier Santander and Curtiss, 2010). In one study, transient colonization by SPtA was observed over a 3-week period, with detection occurring in the spleen, liver, and intestines, while no lethality was observed (Javier Santander and Curtiss, 2010). On the other hand, altered Schaedler flora (ASF) mice have a defined community of eight bacteria that are stably maintained over time and across generations. This ASF microbiota partially ameliorated deficiencies in microbial and host function and, importantly, provides a reduced complexity microbial community compared to conventional microbiota (Wymore Brand et al., 2015; Ott et al., 2020). Moreover, inflammatory conditions *promote the efficient extraintestinal dissemination* of typhoidal *Salmonella* following oral acquisition. *To investigate the impact of temporally induced inflammation*, wild-type (WT) mice were administered dextran sodium sulfate (DSS) prior to inoculation. To evaluate the impact of chronic gastrointestinal inflammation, *IL10^−/−^* mice were used. The goal of the study was to evaluate both male and female WT and *IL10^−/−^* ASF mice as a model for SPtA infection, following oral inoculation. This evaluation involved analyzing several factors, including fecal shedding, weight loss, intestinal colonization, extraintestinal invasion, fluctuations in microbiota, expression of tight junction proteins, and expression of cytokine genes, as well as histopathological scoring. This study investigated whether mice with a defined gut microbiota, specifically the ASF, could serve as a reliable model for SPtA colonization and disease. The findings indicated that the inflammatory state and sex affected colonic colonization of SPtA. Notably, *IL10^−/−^* mice exhibited extraintestinal invasion of SPtA, disruption to the resident microbiota, weight loss, decreases in pro-inflammatory cytokines, and more severe histopathological lesions in cecal tissue.

## 2 Materials and methods

### 2.1 Bacterial strains and inoculation

The human enteric pathogen *Salmonella enterica sub*sp. *enterica* Serovar Paratyphi A (*S.* paratyphi) was used throughout the duration of this experiment. Confirmation of the strain was performed through plating on selective and differential media and molecular typing for cellular antigens and virulence factors as described previously (Hirose et al., 2002). The production of H_2_S and growth on *Enterobacteriaceae* media was confirmed via plating on xylose lysine deoxycholate (XLD) agar (Cat #: R459902, Remel, Lenexa, KS) (Raharjo et al., 2019). Antigen and virulence typing were conducted for the presence and absence of the O, H, and Vi antigen genes *tyv, prt, fliC-d, fliC-a,* and *viaB* by multiplex PCR as described previously (Hirose et al., 2002).

To prepare suspensions for oral gavage of mice, *S.* paratyphi was streaked onto XLD agar, and a single colony was incubated aerobically and statically overnight in 5 mL Luria Bertani (LB) + 0.1% glucose (GLC) at 37 °C. An aliquot (1 mL) of overnight culture was added to 49 mL LB + 0.1% GLC in a flask and incubated with shaking at 180 rotations per minute (RPM) and 37 °C. Incubation continued until the optical density (OD) measured at 600 nm reached 0.8, at which point all 50 mL were centrifuged at 4500 RPM for 15 min at room temperature. Following centrifugation, the supernatant was removed, and the pellet was resuspended in 100 μL of PBS, brought to a final volume of 0.5 mL with PBS, and then added to 4.5 mL of PBS to prepare the final inoculum. The mice were orally gavaged once with an aliquot (0.2 mL, 1 × 10^9^ CFU) (*n* = 6–8 mice per group). The concentration of the inoculum suspension was confirmed through serial dilution and plating on XLD agar.

### 2.2 *In vivo* murine assays

All mouse experiments conducted in this study were approved by the Iowa State University Institutional Animal Use and Care Committee under protocol #IACUC-23-176. Male and female C3H/HeN (2 to 9 months old), 129S6/SvEv WT (3 to 6 months old), and 129S6/SvEv *IL10^−/−^* (2 to 3 months old) mice harboring the ASF-defined microbiota were used for the duration of this study. Breeding and maintenance of the mice were carried out as previously described (Henderson et al., 2015). Mice were housed in flexible film HEPA-filtered isolation caging (Cat #: M-BTM, B0CKLJCQG8 Innovive, San Diego, CA, USA). All handling procedures, including inoculation, sampling, and maintenance of habitat, were performed in a biosafety cabinet using sterile instruments and materials. The mouse diet consisted of Teklad Irradiated Global 19% Protein Extruded Rodent Diet (Cat #: 2919, Inotiv, Bioanalytical Systems, Inc., West Lafayette, IN, USA) and irradiated water (Cat #: M-WB-300C, Innovive, Waples Ct, San Diego, CA). A 12-h light–dark cycle was used. DSS-treated mice received 2% DSS as an additive in their water *ad libitum* starting at 3-DPI and reverted to non-DSS-treated water on the day of inoculation. Collections of the weight and feces of animals took place every 3–4 days. Mice were terminated on 3-, 8-, and 14-DPI by CO_2_ asphyxiation. The study duration was 17 days in total, including 3 days for acclimation and 14 days post-inoculation. For the groups examined at 3- and 8-DPI, there were three males and three females each of WT and *IL10^−/−^* mice, both inoculated and non-inoculated, with the exception of two WT inoculated males at 3-DPI and four *IL10^−/−^* females at 8-DPI. For the groups necropsied at 14-DPI, there were three males and three females of WT and DSS-treated inoculated and non-inoculated C3H/HeN and 129S6/SvEv mice, respectively. Additionally, there were four males and four females of *IL10^−/−^* from the 129S6/SvEv genetic background, both inoculated and non-inoculated. The mice were surface-disinfected using 70% ethanol spray and aseptically opened and sampled in a biosafety cabinet.

### 2.3 Tracking shedding of SPtA in feces, intestinal contents, and immune organs from ASF mice

Fecal samples were aseptically collected every 3–4 days, starting 3-DPI until termination. Fecal shedding was used as a proxy for gastrointestinal colonization of SPtA over time, as reported previously (Ott et al., 2020). At necropsy, ileal, cecal, and colonic contents, as well as the liver and the whole spleen, were aseptically collected to enumerate biogeographical invasion. Quantification involved standardizing tissues to 1 mL with PBS and homogenizing using plastic microcentrifuge pestles (Cat #: 12–141-364, Thermo Fisher Scientific, RRID: SCR_008452). Homogenates were serially diluted and plated on MacConkey agar. Samples demonstrating no growth were enriched by adding 0.5 mL LB + 0.1% glucose and incubating at 225 RPM at 37 °C overnight. Enrichments were plated on MacConkey agar and incubated as before. Tissue enrichments that demonstrated growth were set to half of the limit of quantification (LOQ), while enrichments that demonstrated no growth were set to zero for statistical analysis. LOQ was calculated as the minimum countable range of the volume plated divided by the volume plated in mL multiplied by the dilution factor. SPtA counts were normalized to the original feces, intestinal content, or organ tissue mass and reported as CFU per gram source material.

### 2.4 Histopathology

Ceca tissue was collected at necropsy and stored in 4 mL of 10% neutral buffered formalin for future histological staining. To visualize cellular structures, formalin-fixed tissues were rinsed in 70% ethanol for 1 h and transferred to 4 mL of fresh 70% ethanol prior to staining (Otali et al., 2013). Rinsed tissues were submitted to Iowa State University’s Veterinary Diagnostic Lab for microtome sectioning and hematoxylin and eosin staining. Pathology scoring was performed by a board-certified pathologist at Iowa State University according to the schema described by Suar et al., excluding goblet cell detection (Suar et al., 2006).

### 2.5 RNA extraction and cDNA synthesis

Ceca, colon, and ileal tissues were collected at necropsy and snap-frozen in liquid nitrogen and stored at −80 °C for RNA extraction. Samples were weighed and snap-frozen in liquid nitrogen prior to pulverization in a liquid nitrogen-cooled mortar and micro-pestle (Cat #: H37260-0100, SP Bel-Art, Wayne, NJ, USA) within sterile 1.5 mL microcentrifuge tubes. Total RNA was extracted from ilea samples utilizing the TRIzol reagent (Cat #: 15596026, Thermo Fisher Scientific Inc., Waltham, MA, USA) following the manufacturer’s protocol, purifying the final extracted RNA product from the initial TRIzol extraction with the PureLink RNA Mini Kit (Cat #: 12183018A, Thermo Fisher Scientific Inc., Waltham, MA, USA). Samples were analyzed for purity and initial concentration via a NanoDrop Lite spectrophotometer (ND-LITE, Thermo Fisher Scientific Inc., Waltham, MA, USA). To quantify absolute RNA abundance and quality, the Qubit Broad Range (BR) RNA Assay kit (Cat #: Q10210, Thermo Fisher Scientific Inc., Waltham, MA, USA) was used with a Qubit 4 fluorometer (Cat #: Q33238, Invitrogen, Carlsbad, CA, USA). RNA was removed of contaminating genomic DNA via digestion with RNase-free DNase I (Cat #: 89836, Thermo Fisher Scientific Inc., Waltham, MA, USA) treatment via the manufacturer’s instructions. Extracted mRNA was then converted to cDNA via the High-Capacity cDNA Reverse Transcription Kit (Cat #: 4368813, Thermo Fisher Scientific Inc., Waltham, MA, USA) following the manufacturer’s instructions. The final cDNA was stored at −80 °C for further evaluation.

Total DNA was further extracted from whole mouse ceca (including content) using the TRIzol reagent (Cat #: 15596026, Thermo Fisher Scientific Inc., Waltham, MA, USA) according to the manufacturer’s instructions. Total DNA was quantified using the Qubit 1x BR dsDNA kit (Cat #: Q33230, Thermo Fisher Scientific Inc., Waltham, MA, USA) and diluted to 10 ng/μL in nuclease-free water.

### 2.6 Enumeration of ASF members

ASF strain standard curves were established for ASF members utilizing plasmids previously extracted from *E. coli* DH5 alpha strains with the cloning vector pCR2.1 harboring the 16 s RNA sequences of each ASF member (Supplementary Figure S7, Table 1) (Ott et al., 2020). Plasmid stocks were diluted 5-fold in nuclease-free water and ran in qPCR reactions in triplicate at a standard 95 °C enzyme activation of 2 min, 40 cycles of 95 °C denaturing for 15 s and 60 °C/62 °C annealing/extension for 1 min, and a standard melt curve of 95 °C for 15 s, 60 °C for 1 min, and a 0.1 °C/S climb to 95 °C for 15 s. Annealing/extension temperature of 60 °C was used for ASF 361 (*Lactobacillus murinus*), ASF 492 (*Eubacterium plexicaudatum*), and ASF 500 (*Pseudoflavonifractor* within *Clostridiales*), whereas an annealing/extension temperature of 62 °C was used for ASF 356 (*Clostridia* sp.), ASF 360 (*Lactobacillus intestinalis*), ASF 457 (*Mucispirillum schaedleri*), ASF 502 (*Schaedlerella arabinosiphila*), and ASF 519 (*Parabacteroides goldsteinii*) as reported previously (Wymore Brand et al., 2015; Gomes-Neto et al., 2017; Soh et al., 2019). The Log_10_ genome copies/gram ceca tissue/content extracted from both *IL10^−/−^* and 129S6/SvEv WT ceca gDNA were interpolated using the linear regression for each respective ASF standard curve.

**Table 1:** Primers used for quantitative PCR in this study.

Gene	Primer	Sequence (5′ to 3′)	Annealing TM (°C)
ASF-356 *16 s*	F′	AAAATAATTAGGAGCTTGCTTTTAA	62
	R’	TTAGAAGATGCCTCCTAAGAACC	
ASF-360 *16 s*	F′	GGTGATGACGCTGGGAAC	62
	R’	AAGCAATAGCCATGCAGC	
ASF-361 *16 s*	F′	GAACGAAACTTCTTTATCACC	60
	R’	TAGCATAGCCACCTTTTACA	
ASF-457 *16 s*	F′	TCTCTTCGGGGATGATTAAAC	62
	R’	AACTTTTCCTATATAAACATGCAC	
ASF-492 *16 s*	F′	AATTCCTTCGGGGAGGAAGC	60
	R’	TAAAACCATGCGGTTTTAAAAAC	
ASF-500 *16 s*	F′	ACGGAGGACCCCTGAAGG	60
	R’	AGCGATAAATCTTTGATGTCC	
ASF-502 *16 s*	F′	GAGCGAAGCACTTTTTTAGAAC	62
	R’	TTACACCACCTCAGTTTTTACC	
ASF-519 *16 s*	F′	GCAGCACGATGTAGCAATACA	62
	R’	TTAACAAATATTTCCATGTGGAAC	
*GUSB*	F′	CCGATTATCCAGAGCGAGTATG	60
	R’	CTCAGCGGTGACTGGTTCG	
*CLDN-1*	F′	TCTACGAGGGACTGTGGATG	60
	R’	TCAGATTCAGCAAGGAGTCG	
*CLDN-2*	F′	CCTCGCTGGCTTGTATTATCTCTG	60
	R’	GAGTAGAAGTCCCGAAGGA	
*ZO-1*	F′	AGCTCATAGTTCAACACAGCCTCCAG	60
	R’	TTCTTCCACAGCTGAAGGACTCACAG	
*OCLN*	F′	AGAGGCTATGGGACAGGGCTCTTTGG	60
	R’	CCAACAGGAAGCCTTTGGCTGCTCTTGG	
*MEFV*	F′	AGGCTTCAAGGACTTTACAACAA	60
	R’	TCATGCGAATGAGACTCCCA	
*TNFα*	F′	CCACCACGCTCTTCTGTCTA	60
	R’	AGGGTCTGGGCCATAGAACT	
IFNγ	F′	AGCGGCTGACTGAACTCAGATTGT	63
	R’	GTCACAGTTTTCAGCTGTATAGGG	
*IL6*	F′	GTTCTCTGGGAAATCGTGGA	54
	R’	TGTACTCCAGGTAGCTATGG	
*IL10*	F′	ATGCAGGACTTTAAGGGTTACTTG	54
	R’	AGACACCTTGGTCTTGGAGCTTA	

Linear regressions were generated from plotted values of C_t_ to a known number of gene copies in each dilution of the standard curve samples, excluding the highest dilution for some due to high C_t_ variation (ASF 356, ASF 502, and ASF 519). DNA isolated from mouse ceca content was utilized in qPCR under the same conditions and fit to the linear regression to enumerate Log_10_ genome copies of each taxon. LOQ for ASF quantification data was calculated by calculating Log_10_(16 s copies/g) using the intercept value of the calculated regression equation as the input ct value, accounting for the highest sample tau value and highest sample mass.

### 2.7 Quantification of gene expression via qPCR

The relative expression of host inflammation and tight junction genes was evaluated via qPCR analysis of generated cDNA. In brief, 10 ng template cDNA was used in qPCR to analyze beta-glucuronidase (GUSB) expression as a housekeeping gene (Gong et al., 2016). Tight junction protein-encoding genes for occludin (*Ocln*), claudin-1 (*Cldn-1*), claudin-2 (*Cldn-2*), and zonula occludens-1 (*ZO-1*) were observed in cecal cDNA using qPCR (Table 1). Cytokine protein-encoding genes for tumor necrosis factor alpha *(TNFα)*, interferon gamma (*IFNγ*), interleukin 6 (*IL6*), and interleukin 10 (*IL10*) were assessed in ileal cDNA through RTqPCR. The primers and genes targeted are described in Table 1. The qPCR reactions were performed using the PowerTrack SYBR Green qPCR Master Mix (Cat #: A46109, Thermo Fisher Scientific Inc., Waltham, MA, USA) according to the manufacturer’s instructions. The following reaction conditions were applied; one cycle of 10 min at 95 °C for enzyme activation, 40 cycles of (1) 15 s at 95 °C for DNA denaturation, and (2) 1 min at 60 °C or 62 °C for both annealing and extension, followed by a single cycle melt curve of 15 s at 95 °C, 1 min at 60 °C, and a ramp of 0.1 °C/S to 95 °C. The 2^-ΔΔct^ method was used to evaluate changes in gene expression between treated and untreated groups (Redweik et al., 2021). The geometric mean of each sample’s expression of the housekeeping gene GUSB was used as the normalization index.

### 2.8 Statistics

**Figure 1:**
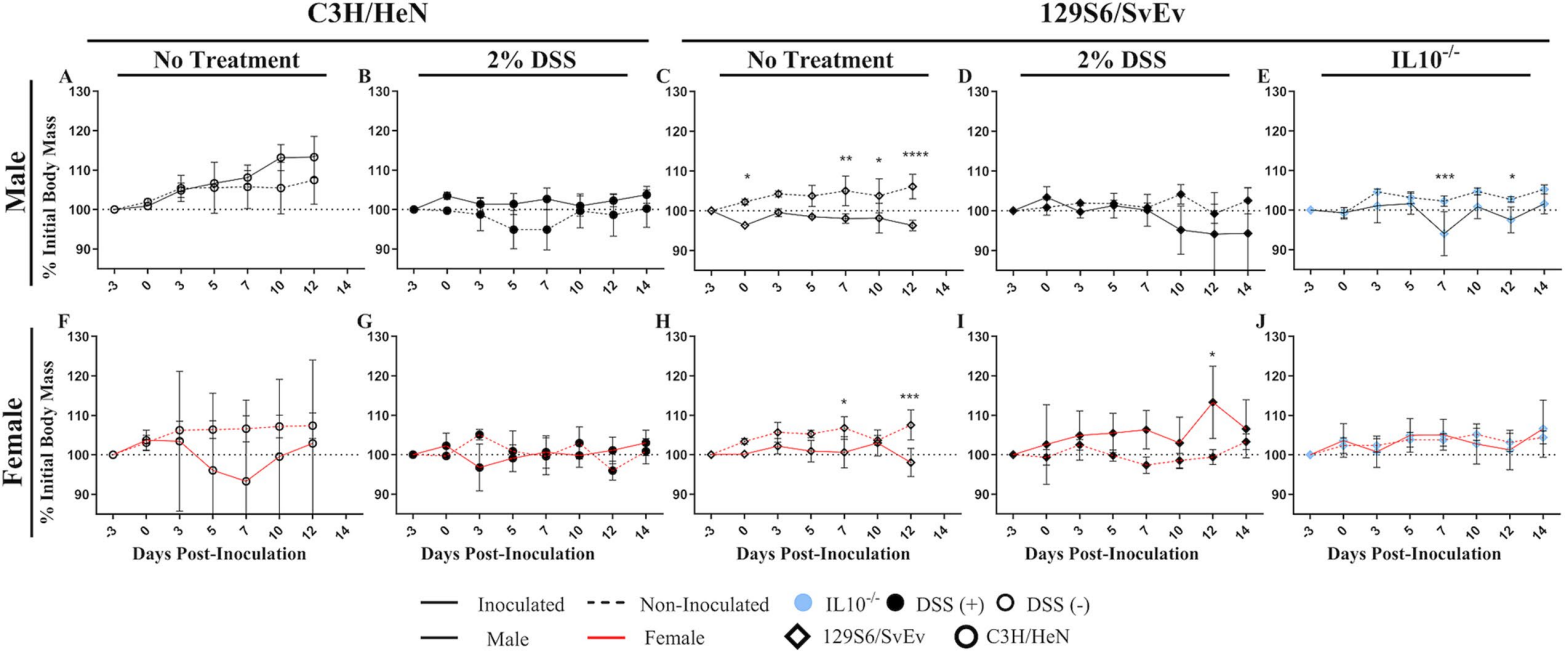
Effect of inoculation with SPtA on change in weight. Change in weight respective to initial body weight of male and female of either C3H/HeN or 129S6/SvEv mice provided water with or without 2% DSS. Comparisons drawn between inoculated and non-inoculated **A.** C3H/HeN non-treated males, **B.** C3H/HeN DSS-treated males, **C.** 129S6/SvEv non-treated males, **D.** 129S6/SvEv DSS-treated males, **E.** 129S6/SvEv *IL10^−/−^* males, **F.** C3H/HeN non-treated females, **G.** C3H/HeN DSS-treated females, **H.** 129S6/SvEv non-treated females, **I.** 129S6/SvEv DSS-treated females, and **J.** 129S6/SvEv *IL10^−/−^* females. DSS, dextran sodium sulfate; *IL10*, interleukin 10 *n* = 3 mice per group at each time point, *n* = 4 *IL10^−/−^* mice per group at each time point. *, *p*-value < 0.05; **, *p*-value < 0.01; ***, *p*-value < 0.001; ****, *p*-value < 0.0001.

**Figure 2:**
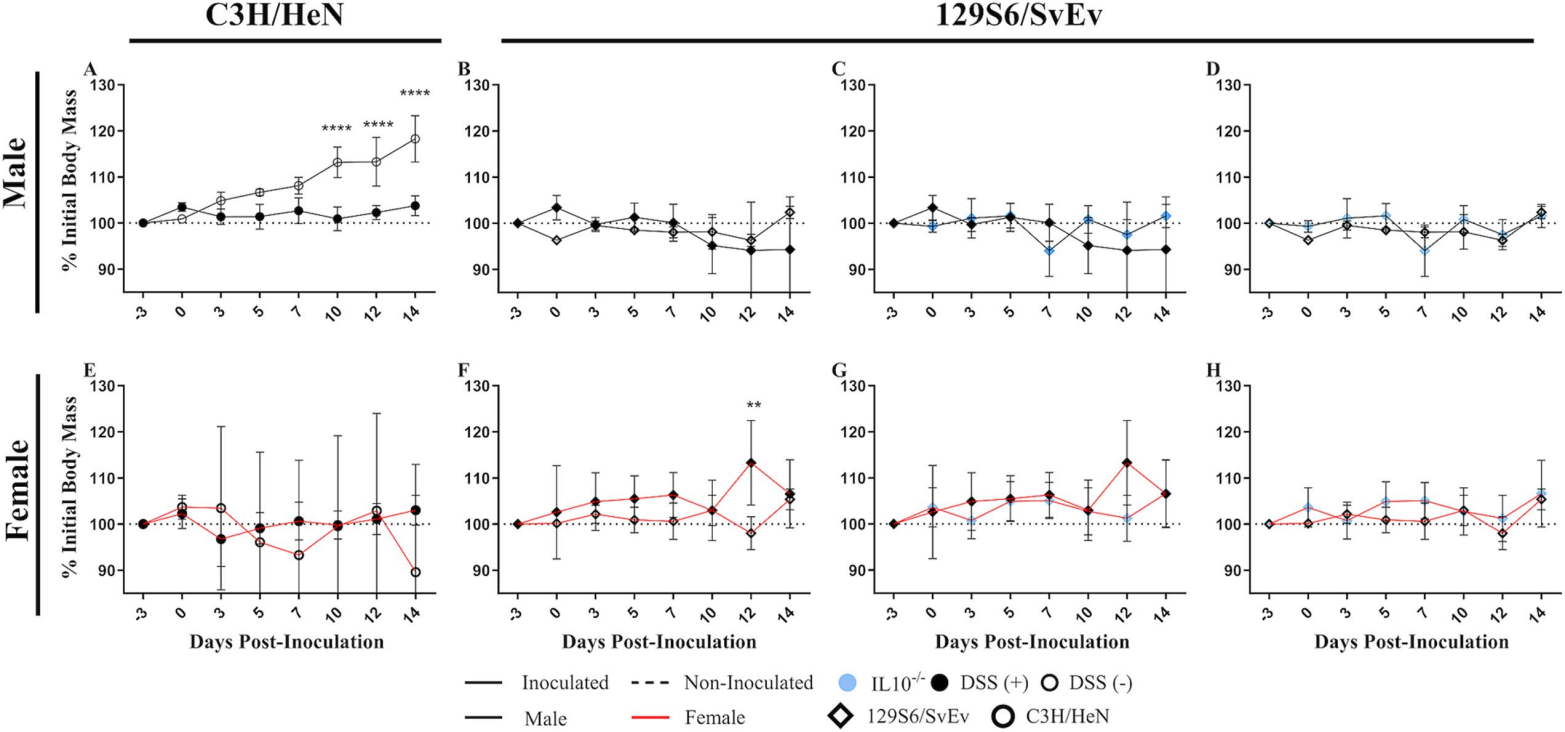
Effect of inflammatory conditions on SPtA fecal shedding. Change in weight respective to initial body weight of male and female C3H/HeN or 129S6/SvEv mice either treated with 2% DSS or untreated irradiated water. Comparisons drawn between inoculated **A.** C3H/HeN DSS- and non-treated males, **B.** 129S6/SvEv DSS- and non-treated males, **C.** 129S6/SSvEv DSS-treated and *IL10^−/−^* males, **D.** 129S6/SvEv non-treated and *IL10^−/−^* males, **E.** C3H/HeN DSS- and non-treated females, **F.** 129S6/SvEv DSS- and non-treated females, **G.** 129S6/SSvEv DSS-treated and *IL10^−/−^* females, and **H.** 129S6/SvEv non-treated and *IL10^−/−^* females. DSS, dextran sodium sulfate; *IL10*, interleukin 10. *n* = 3 mice per group at each time point, *n* = 4 *IL10^−/−^* mice per group at each time point. *, *p*-value < 0.05; **, *p*-value < 0.01; ***, *p*-value < 0.001; ****, *p*-value < 0.0001.

**Figure 3:**
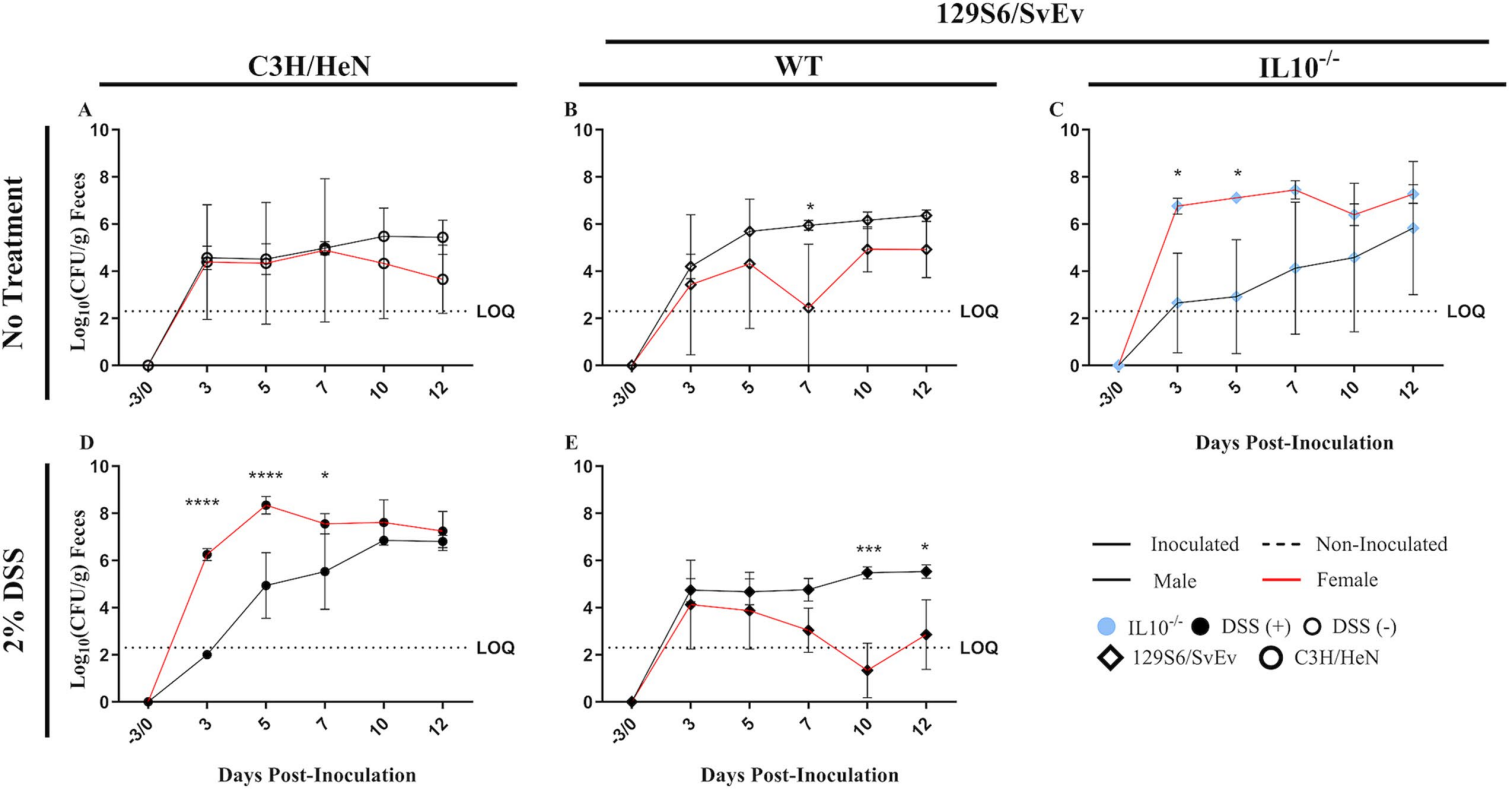
Effect of sex on SPtA fecal shedding. Log_10_ (CFU/mL) *S.* paratyphi A detected in the feces of male and female C3H/HeN or 129S6/SvEv mice that were treated with or without 2% DSS in the drinking water. Non-zero values below the LOQ (intestinal content = 2.30103, organs = 1.60206) represent enriched positives valued at Log_10_(1/2 LOQ). Comparisons drawn between male and female **A.** C3H/HeN non-treated mice, **B.** 129S6/SvEv non-treated mice, **C.** 129S6/SvEv *IL10^−/−^* mice, **D.** C3H/HeN DSS-treated mice, and **E.** 129S6/SvEv DSS-treated mice. DSS, dextran sodium sulfate; *IL10*, interleukin 10. *n* = 3 mice per group at each time point, *n* = 4 *IL10^−/−^* mice per group at each time point. *, *p*-value < 0.05; **, *p*-value < 0.01; ***, *p*-value < 0.001; ****, *p*-value < 0.0001.

**Figure 4:**
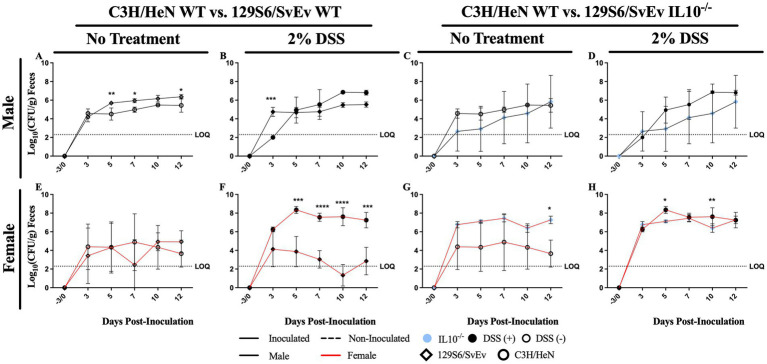
Effects of genetic background on SPtA fecal shedding. Log_10_ (CFU/mL) *S.* paratyphi A detected in the feces of male and female mice of either C3H/HeN or 129S6/SvEv genetic background either treated with 2% DSS or untouched irradiated water. Nonzero values below the LOQ (intestinal content = 2.30103, organs = 1.60206) represent enriched positives valued at Log_10_(1/2 LOQ). Comparisons drawn between C3H/HeN and 129S6/SvEv **A.** non-treated males **B.** DSS-treated males, **C.** non-treated and *IL10^−/−^* males respectively, **D.** DSS-treated and *IL10^−/−^* males respectively, **E.** non-treated females, **F.** DSS-treated females, **E.** non-treated and *IL10^−/−^* females respectively, and **G.** DSS-treated and *IL10^−/−^* females respectively. **H.** DSS-treated and IL10-/- females respectively. DSS, dextran sodium sulfate; *IL10*, interleukin 10. *n* = 3 mice per group at each time point, *n* = 4 *IL10^−/−^* mice per group at each time point. *, *p*-value < 0.05; **, *p*-value < 0.01; ***, *p*-value < 0.001; ****, *p*-value < 0.0001.

**Figure 5:**
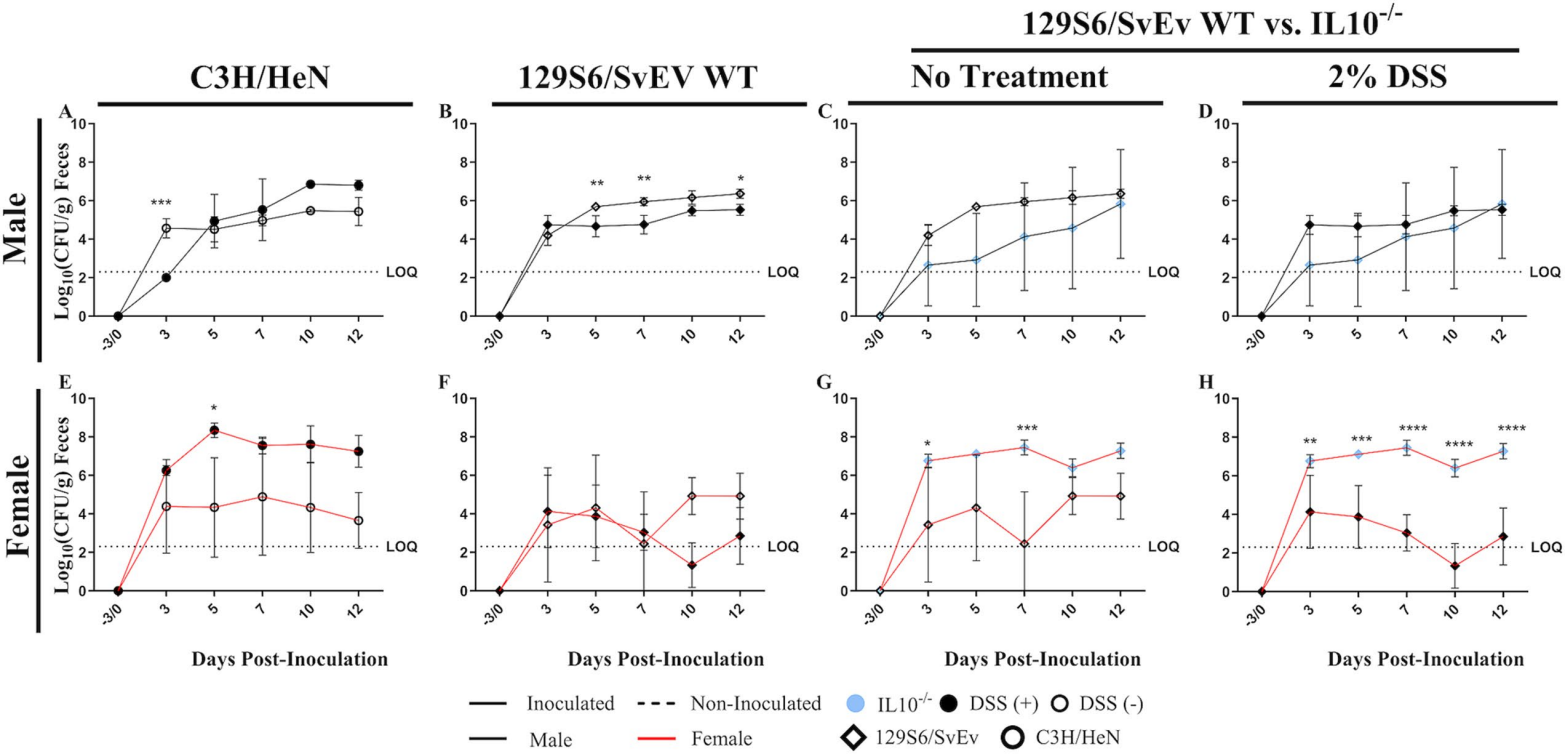
Effect of inflammatory conditions on SPtA fecal shedding. Log_10_ (CFU/mL) *S.* paratyphi A detected in the feces of male and female mice of either C3H/HeN or 129S6/SvEv genetic background either treated with 2% DSS or untouched irradiated water. Non-zero values below the LOQ (intestinal content = 2.30103, organs = 1.60206) represent enriched positives valued at Log_10_(1/2 LOQ). Comparisons drawn between **A.** C3H/HeN DSS- and non-treated males, **B.** 129S6/SvEv DSS-and non-treated males, **C.** 129S6/SvEv *IL10^−/−^* and non-treated males, **D.** 129S6/SvEv *IL10^−/−^* and DSS-treated males, **E.** C3H/HeN DSS- and non-treated females, **F.** 129S6/SvEv DSS-and non-treated females, **G.** 129S6/SvEv *IL10^−/−^* and non-treated females, and **H.** 129S6/SvEv *IL10^−/−^* and DSS-treated females. DSS, dextran sodium sulfate; *IL10*, interleukin 10. *n* = 3 mice per group at each time point, *n* = 4 *IL10^−/−^* mice per group at each time point. *, *p*-value < 0.05; **, *p*-value < 0.01; ***, *p*-value < 0.001; ****, *p*-value < 0.0001.

**Figure 6:**
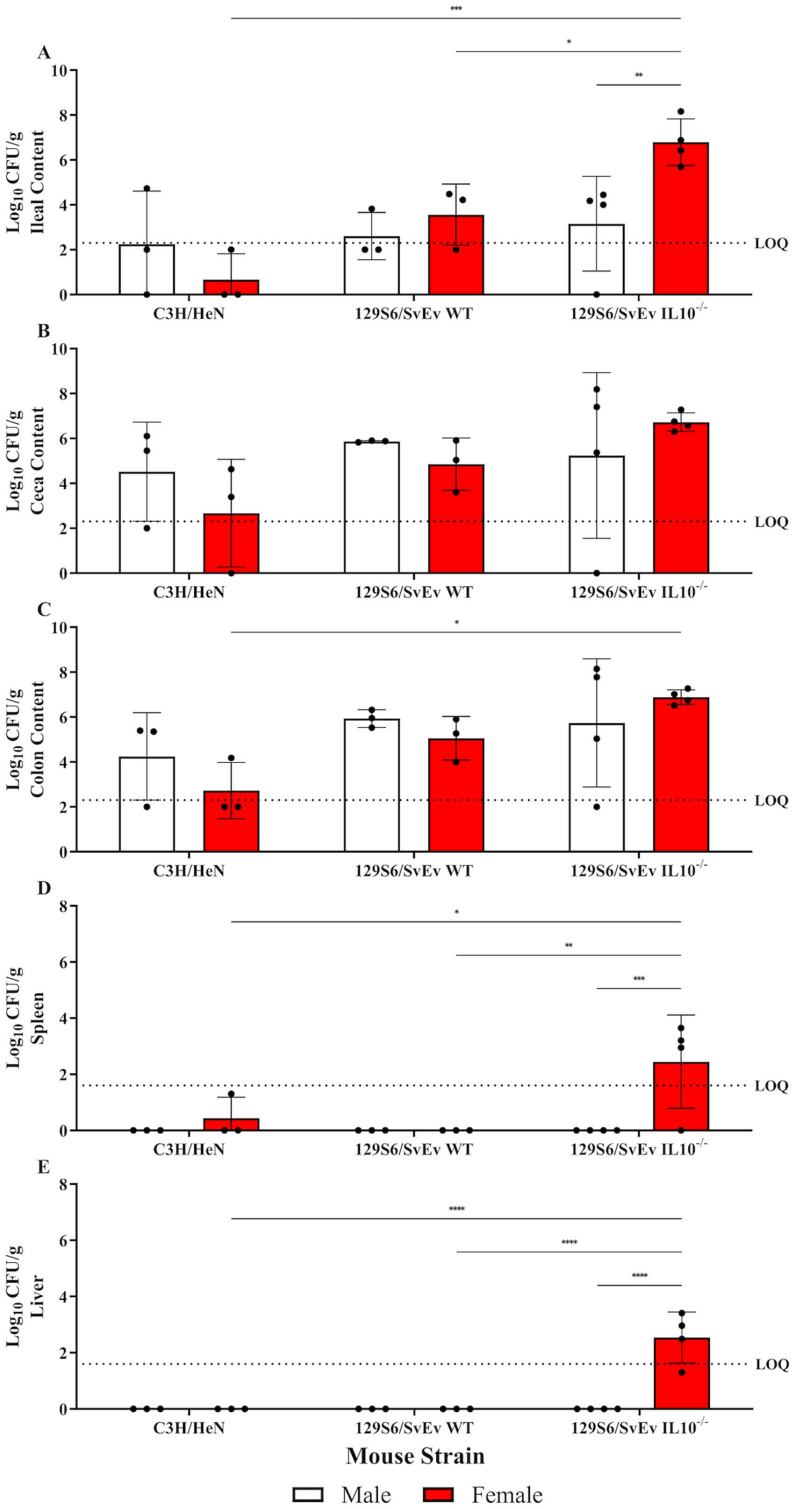
Effect of sex and genetic background on SPtA colonization. Log10 (CFU/mL) *S.* paratyphi A detected in intestinal content and extraintestinal tissues of non-DSS-treated male and female mice of C3H/HeN, 129S6/SvEv WT, or 129S6/SvEv *IL10^−/−^* genetic background 14 DPI. Non-zero values below the LOQ (intestinal content = 2.30103, organs = 1.60206) represent enriched positives valued at Log_10_(1/2 LOQ). Samples tested include **A.** ileal content, **B.** ceca content, **C.** colon content, **D.** spleen, and **E.** liver. DSS, dextran sodium sulfate; *IL10*, interleukin 10. *n* = 3 mice per group, *n* = 4 *IL10^−/−^* mice per group. *, *p*-value < 0.05; **, *p*-value < 0.01; ***, *p*-value < 0.001; ****, *p*-value < 0.0001.

**Figure 7:**
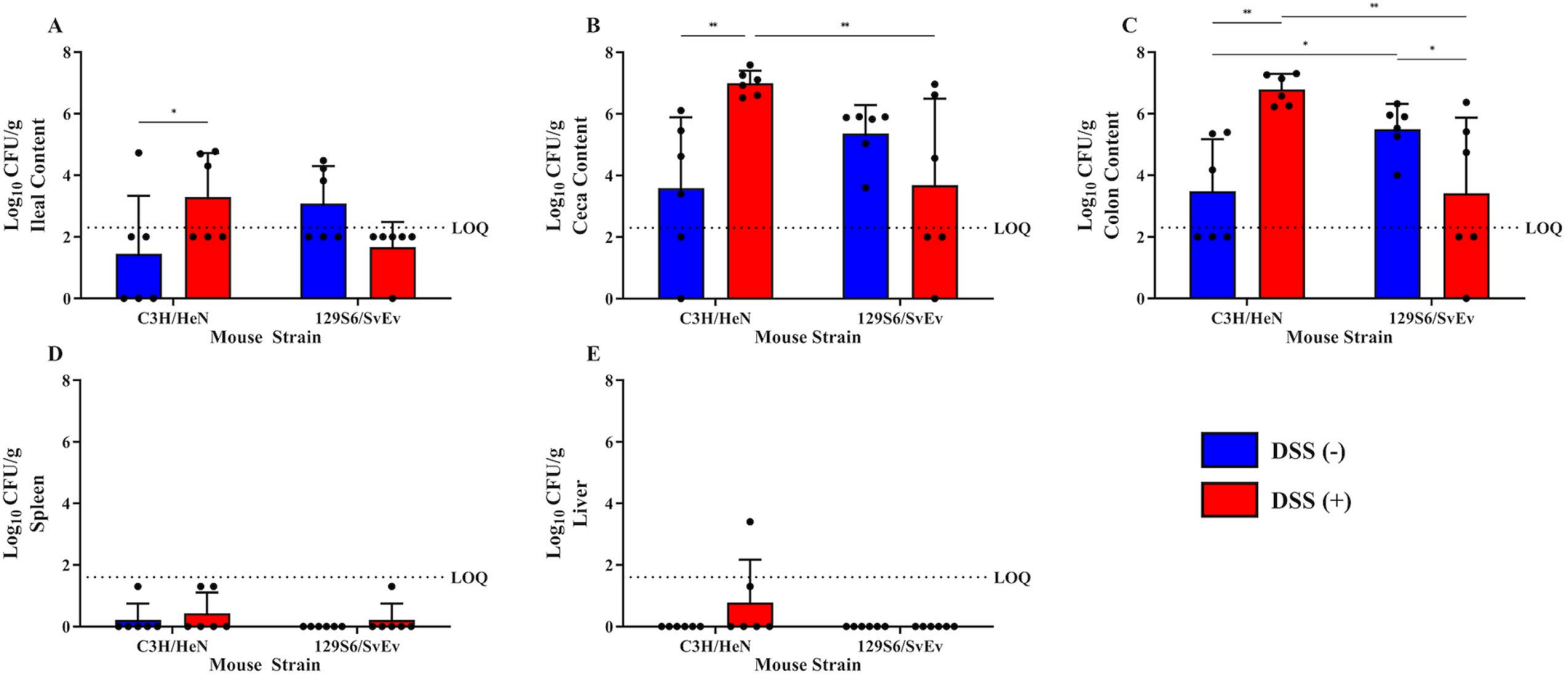
Effect of DSS and genetic background on SPtA colonization. Log_10_ (CFU/mL) *S.* paratyphi A detected in intestinal content and extraintestinal tissues at necropsy of DSS-treated male and female mice of either C3H/HeN or 129S6/SvEv WT genetic background 14 DPI. Non-zero values below the LOQ (intestinal content = 2.30103, organs = 1.60206) represent enriched positives valued at Log_10_(1/2 LOQ). Samples tested include **A.** ileal content, **B.** ceca content, **C.** colon content, **D.** spleen, and **E.** liver. DSS, dextran sodium sulfate; *IL10*, interleukin 10. *n* = 3 male and female mice per group, respectively. *, *p*-value < 0.05; **, *p*-value < 0.01; ***, *p*-value < 0.001; ****, *p*-value < 0.0001.

**Figure 8:**
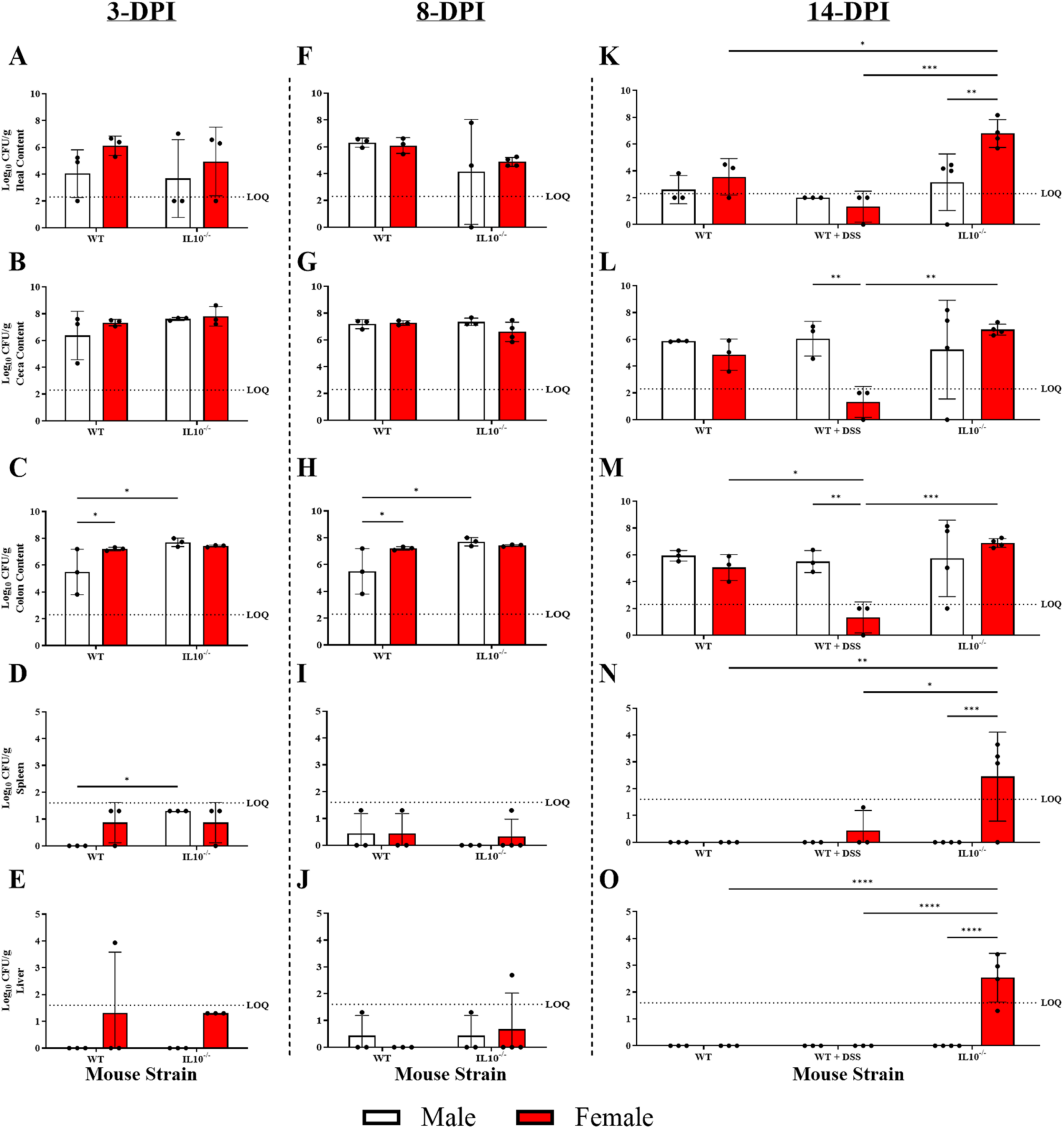
Effect of sex and inflammatory conditions on SPtA colonization 14-DPI. Log10 (CFU/mL) *S.* paratyphi A detected in intestinal content and extraintestinal tissues at necropsy of male and female mice of either non-DSS-treated 129S6/SvEv WT, DSS-treated 129S6/SvEv WT, or 129S6/SvEv *IL10^−/−^* genetic background 3-, 8-, and 14-DPI. Non-zero values below the LOQ (intestinal content = 2.30103, organs = 1.60206) represent enriched positives valued at Log_10_(1/2 LOQ). Samples tested include **A, F, K.** ileal content, **B, G, L.** ceca content, **C. H. M.** colon content, **D, I, N.** spleen, and **E, J, O.** liver. *IL10*, interleukin 10. 3-DPI *n* = 3 mice per group. 8-DPI *n* = 3 mice per group, *n* = 4 female *IL10^−/−^* mice. 14-DPI *n* = 3 mice per group, *n* = 4 *IL10^−/−^* mice per group. *IL10*, interleukin 10. *, *p*-value < 0.05; **, *p*-value < 0.01; ***, *p*-value < 0.001; ****, *p*-value < 0.0001.

**Figure 9:**
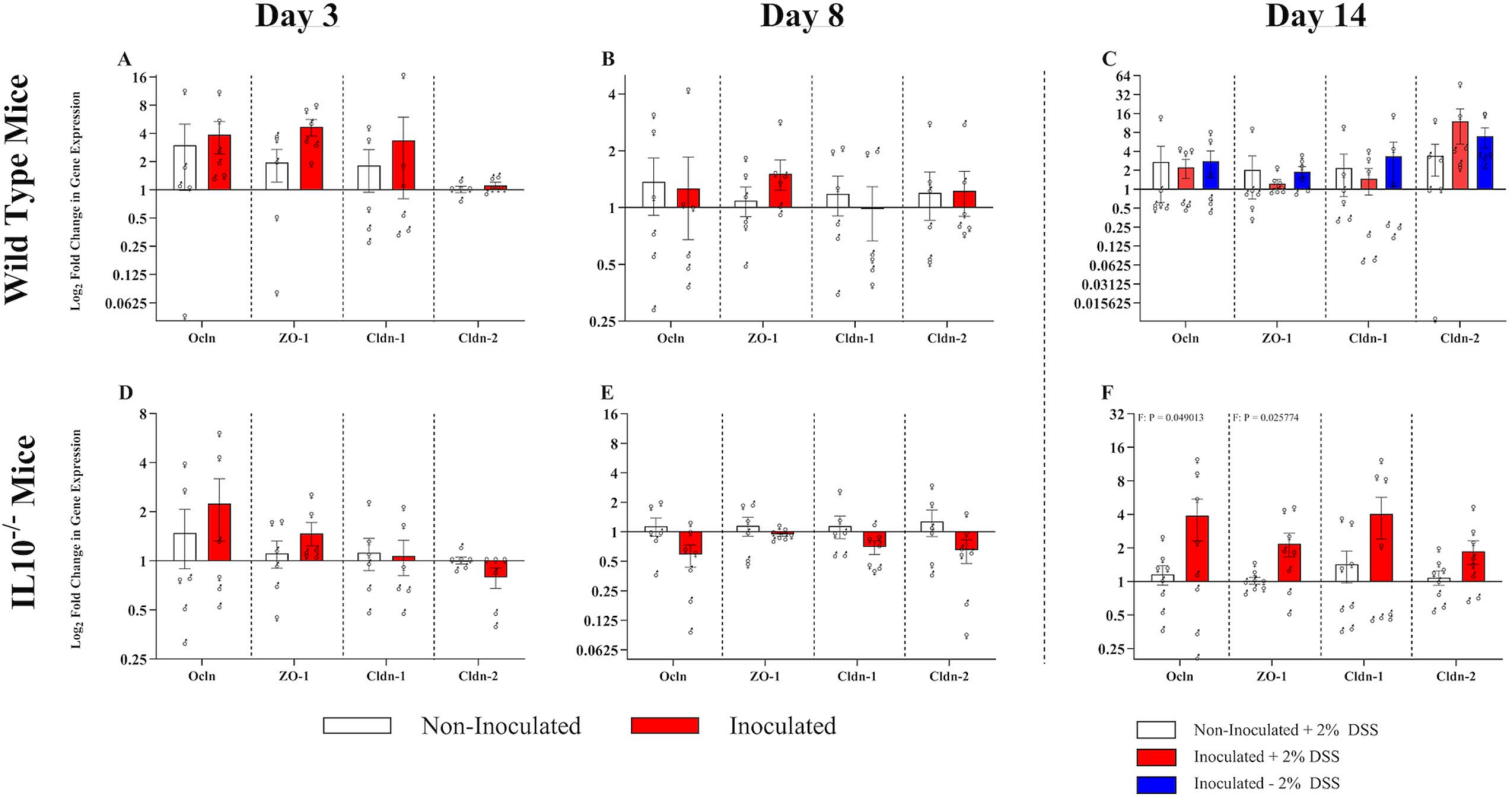
Effect of SPtA inoculation on tight junction protein expression. Relative 2^-ΔΔCT^ expression of tight junction proteins in ceca cDNA extracted from non-DSS-treated 129S6/SvEv WT, DSS-treated 129S6/SvEv WT, or 129S6/SvEv *IL10^−/−^* inoculated with *S.* paratyphi or PBS 3-, 8-, or 14-DPI. qPCR was run in duplicate. Comparisons drawn between inoculated and non-inoculated **A.** WT mice 3-DPI, **B.** WT mice 8-DPI, **C.** WT mice 14-DPI, **D.** *IL10^−/−^* mice 3-DPI, **E.** *IL10^−/−^* 8-DPI, and **F.** *IL10^−/−^* mice 14-DPI. *Ocln*, occludin; *ZO-1*, zonula occludens-1; *Cldn-1*, claudin-1; *Cldn-2*, claudin-2. *n* = 3 WT mice per males and females, respectively, per group at all timepoints excepting *n* = 2 WT non-inoculated males at 3-DPI. *n* = 3 *IL10^−/−^* mice per males and females, respectively, per group at 3- and 8-DPI excepting *n* = 4 *IL10^−/−^* inoculated females at 8-DPI. *n* = 4 *IL10^−/−^* mice per males and females, respectively, per group at 14-DPI. Sex-independent *p*-values reported below relevant groups. *, *p*-value < 0.05; **, *p*-value < 0.01; ***, *p*-value < 0.001; ****, *p*-value < 0.0001.

**Figure 10:**
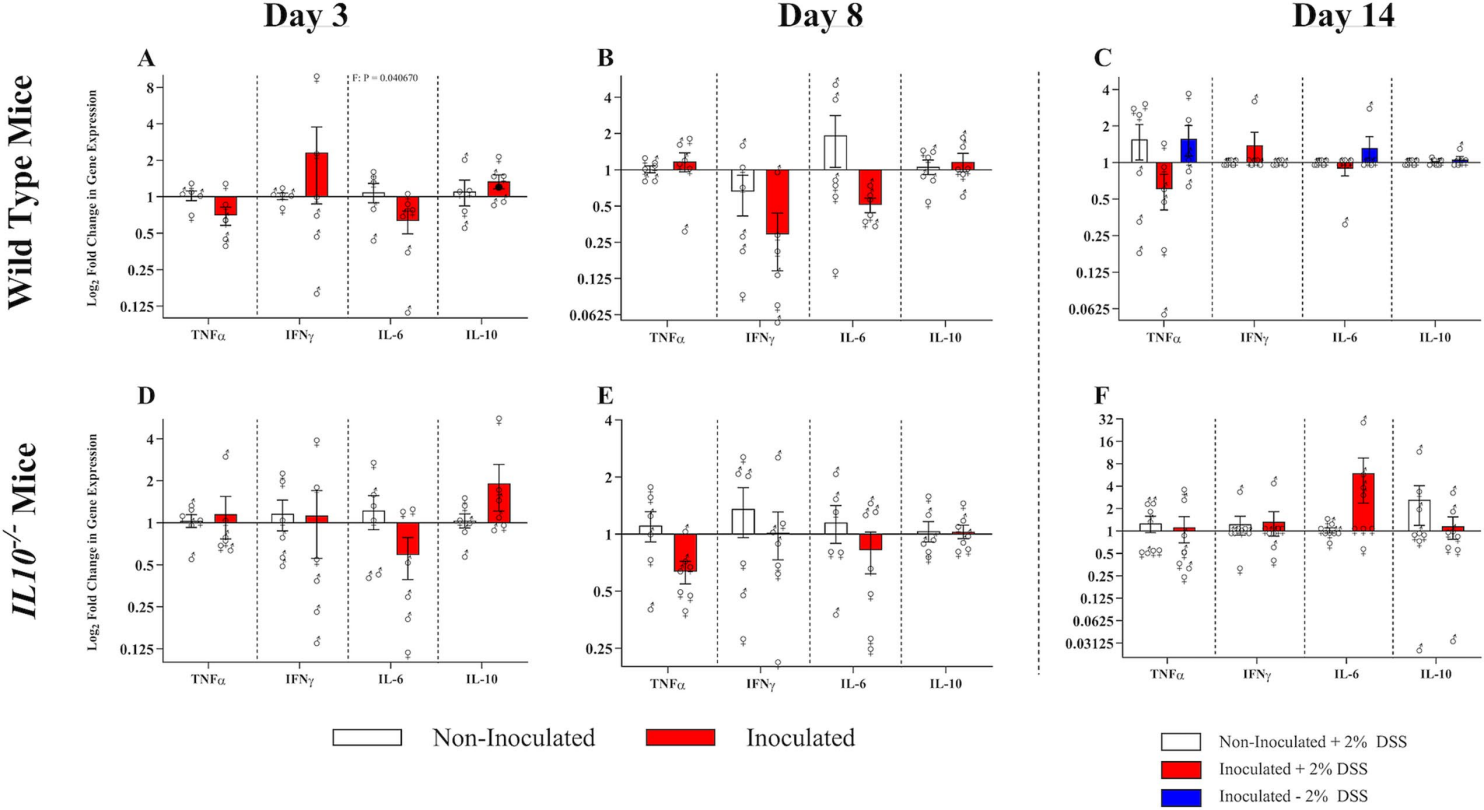
Effect of SPtA inoculation on ileal cytokine expression. Relative 2^-ΔΔCT^ expression of cytokine proteins in ileal cDNA extracted from non-DSS-treated 129S6/SvEv WT, DSS-treated 129S6/SvEv WT, or 129S6/SvEv *IL10^−/−^* inoculated with *S.* paratyphi or PBS 3-, 8-, or 14-DPI. qPCR was run in duplicate. Sex-independent p-values reported below relevant groups. Comparisons drawn between inoculated and non-inoculated **A.** WT mice 3-DPI, **B.** WT mice 8-DPI, **C.** WT mice 14-DPI, **D.** *IL10^−/−^* mice 3-DPI, **E.** *IL10^−/−^* 8-DPI, and **F.** *IL10^−/−^* mice 14-DPI. *TNFα*, tumor necrosis factor alpha; *IFNγ*, interferon gamma; *IL6*, interleukin 6; *IL10*, interleukin 10. *n* = 3 WT mice per males and females, respectively, per group at all timepoints excepting *n* = 2 WT non-inoculated males at 3-DPI. *n* = 3 *IL10^−/−^* mice per males and females, respectively, per group at 3- and 8-DPI excepting *n* = 4 *IL10^−/−^* inoculated females at 8-DPI. *n* = 4 *IL10^−/−^* mice per males and females, respectively, per group at 14-DPI. *, *p*-value < 0.05; **, *p*-value < 0.01; ***, *p*-value < 0.001; ****, *p*-value < 0.0001.

**Figure 11:**
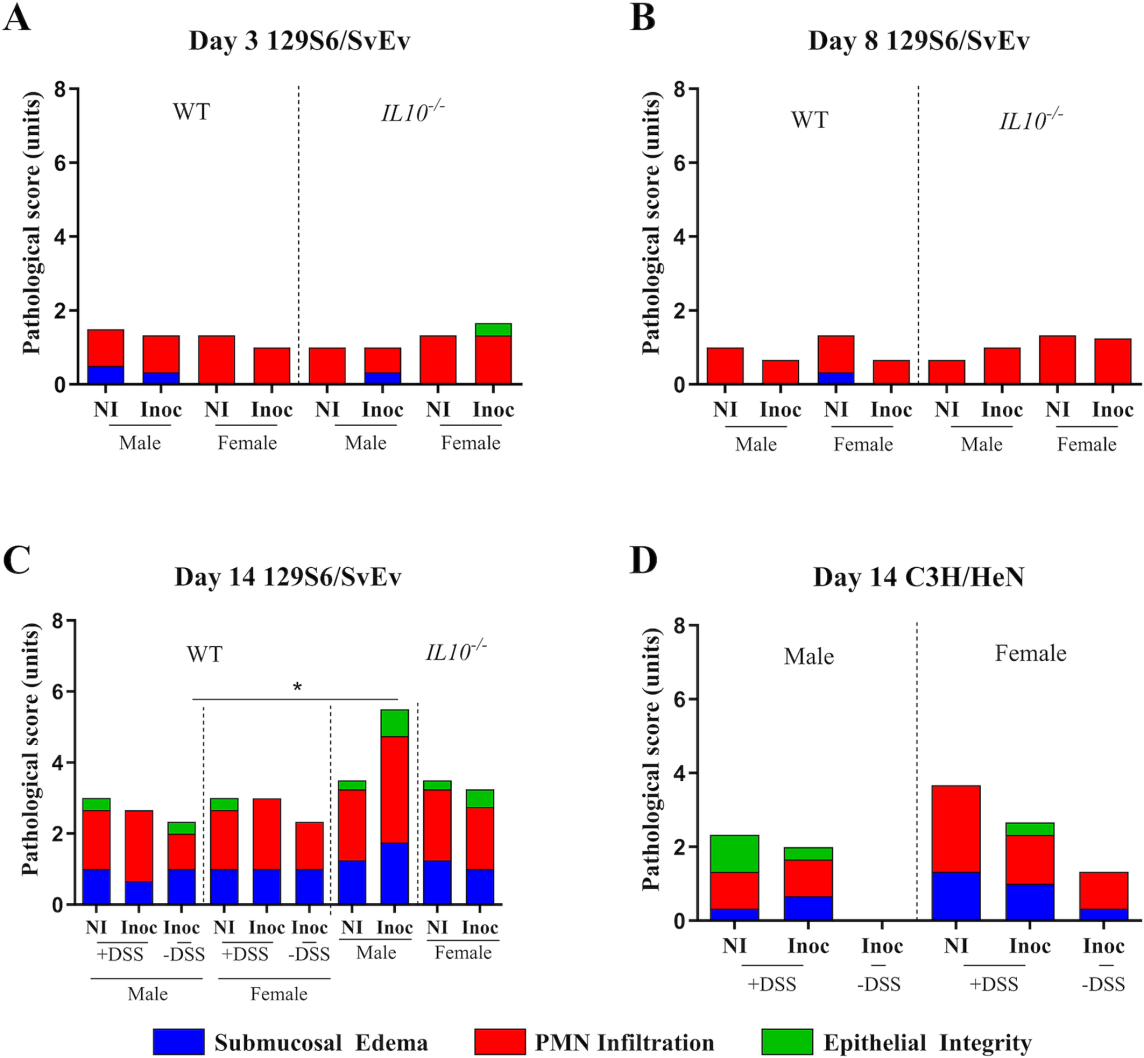
Effect of inflammatory conditions on cecal pathology. Pathological scoring of H&E stained ceca tissues quantified as a combination of submucosal edema (0—no pathological changes, 1—detectable edema [submucosal edema, <10%], 2—moderate edema [submucosal edema, 10 to 40%], and 3—profound edema [submucosal edema, ≥40%]), PMN infiltration into the lamina propria (0—fewer than 5 PMN per high-power field, 1—5 to 20 PMN per high-power field, 2—21 to 60 PMN per high-power field, 3—61 to 100 PMN per high-power field, and 4—more than 100 PMN per high-power field), and epithelial integrity (0—no pathological changes, 1—epithelial desquamation, 2—erosion of the epithelial surface, and 3—epithelial ulceration). Statistical testing is done according to mouse group between treatments. Analysis was performed on all tested mice tissues isolated at necropsy. A certified pathologist performed analysis. Comparisons drawn between **A.** 129S6/SvEv mice 3-DPI, **B.** 129S6/SvEv mice 8-DPI, **C.** 129S6/SvEv mice 14-DPI, and **D.** C3H/HeN mice 14-DPI. *IL10*, interleukin 10. *n* = 3 WT mice per males and females, respectively, per group at all timepoints excepting *n* = 2 WT non-inoculated males at 3-DPI. *n* = 3 *IL10^−/−^* mice per males and females, respectively, per group at 3- and 8-DPI excepting *n* = 4 *IL10^−/−^* inoculated females at 8-DPI. *n* = 4 *IL10^−/−^* mice per males and females, respectively, per group at 14-DPI. *, *p*-value < 0.05; **, *p*-value < 0.01; ***, *p*-value < 0.001; ****, *p*-value < 0.0001.

**Figure 12:**
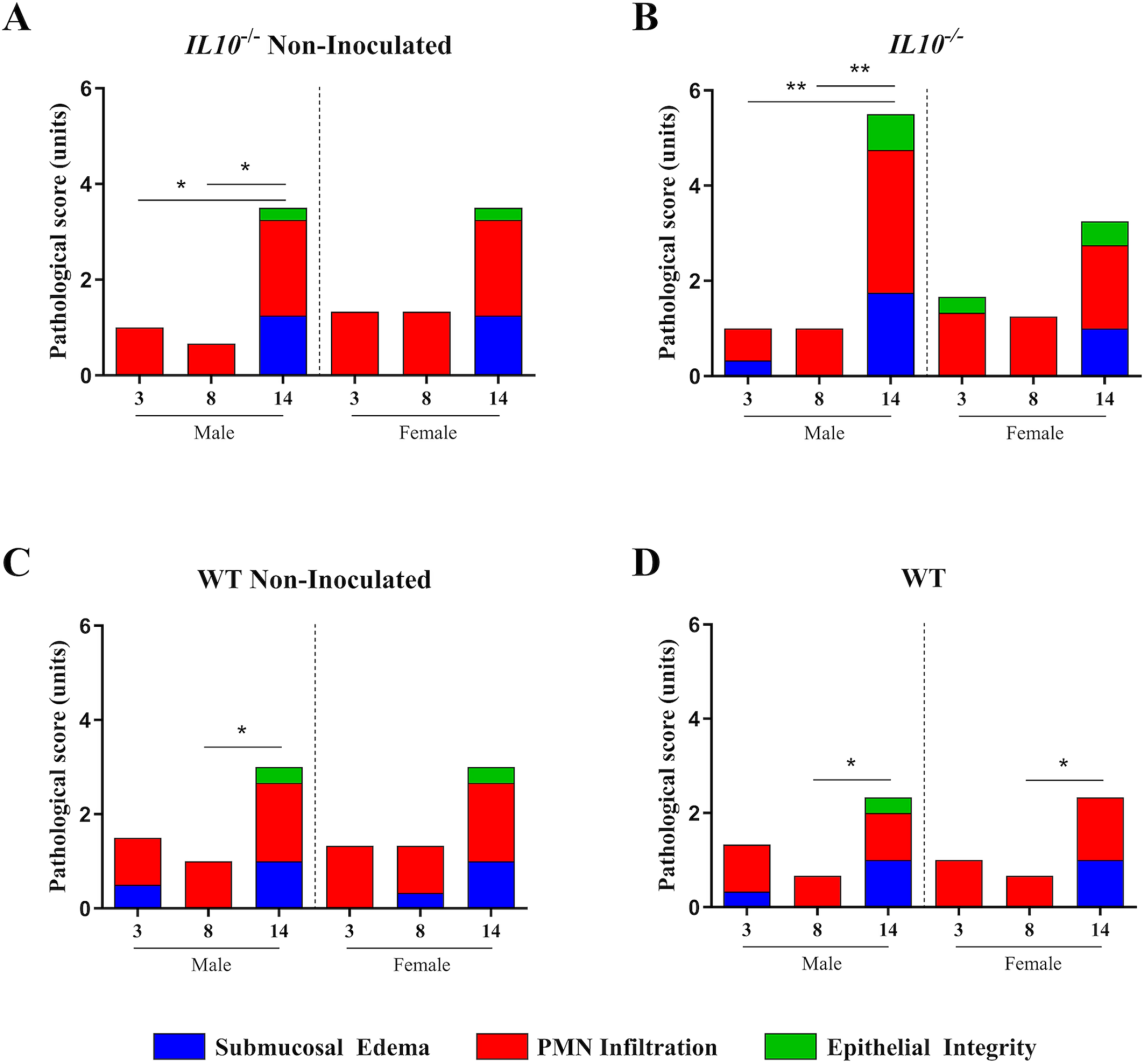
Effect of time post-inoculation on cecal pathology. Pathological scoring of H&E stained ceca tissues quantified as a combination of submucosal edema (0—no pathological changes, 1—detectable edema [submucosal edema, <10%], 2—moderate edema [submucosal edema, 10 to 40%], and 3—profound edema [submucosal edema, ≥40%]), PMN infiltration into the lamina propria (0—fewer than 5 PMN per high-power field, 1—5 to 20 PMN per high-power field, 2—21 to 60 PMN per high-power field, 3—61 to 100 PMN per high-power field, and 4—more than 100 PMN per high-power field), and epithelial integrity (0—no pathological changes, 1—epithelial desquamation, 2—erosion of the epithelial surface, and 3—epithelial ulceration). Statistical testing is done according to mouse group between times. Analysis was performed on all tested mice tissues isolated at necropsy. A certified pathologist performed analysis. Comparisons drawn between 129S6/SvEv **A.** *IL10^−/−^* non-inoculated mice, **B.** *IL10^−/−^* inoculated mice, **C.** WT non-inoculated mice, and **D.** inoculated mice. *IL10*, interleukin 10. *n* = 3 WT mice per males and females, respectively, per group at all timepoints excepting *n* = 2 WT non-inoculated males at 3-DPI. *n* = 3 *IL10^−/−^* mice per males and females, respectively, per group at 3- and 8-DPI excepting *n* = 4 *IL10^−/−^* inoculated females at 8-DPI. *n* = 4 *IL10^−/−^* mice per males and females, respectively, per group at 14-DPI. *, *p*-value < 0.05; **, *p*-value < 0.01; ***, *p*-value < 0.001; ****, *p*-value < 0.0001.

**Figure 13:**
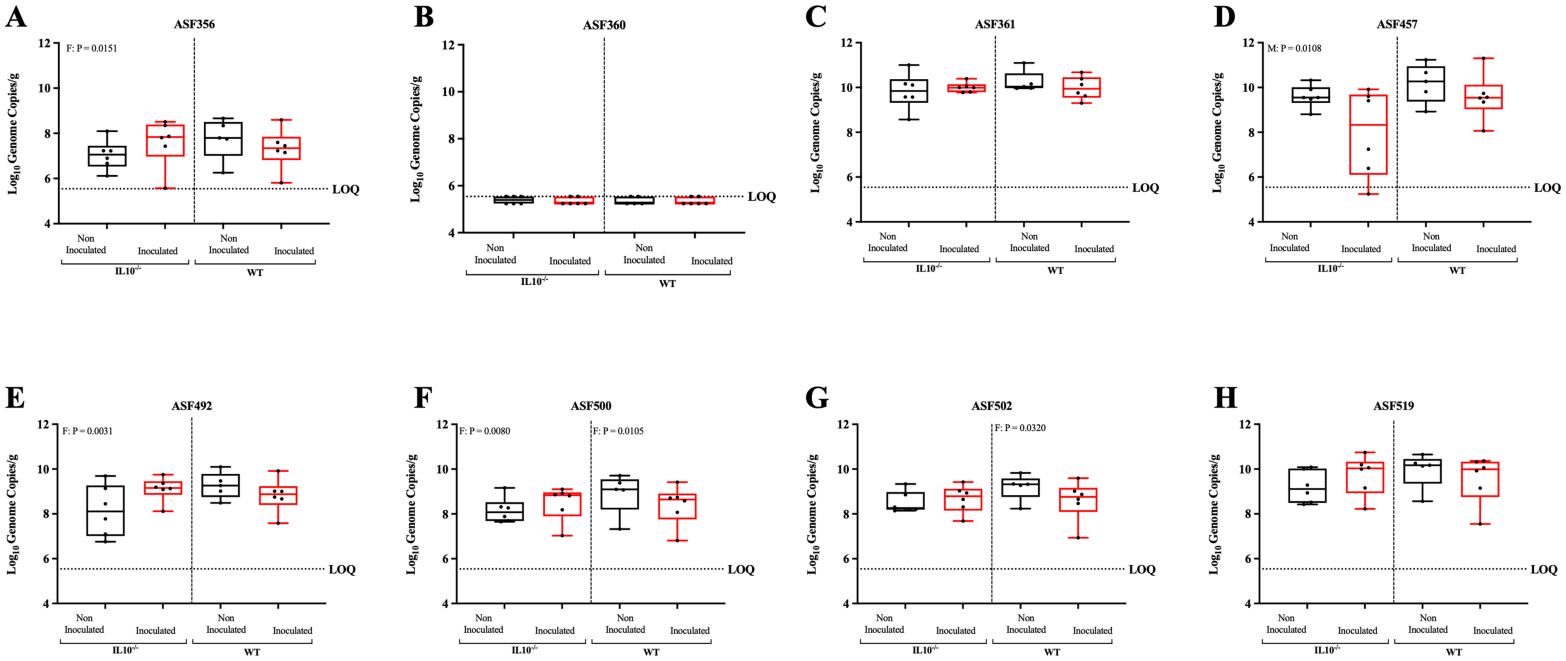
Effect of SPtA inoculation on resident microbiota 3-DPI. Log_10_ Genome Copies of each ASF member per gram ceca tissue extracted from detected in 129S6/SvEv WT and 129S6/SvEv *IL10^−/−^* genetic background either inoculated with *S.* paratyphi or PBS at 3 DPI. Ct values for each primer pair were fit to a linear regression generated using dilutions of cloning vectors containing the 16 s rRNA gene of each member (Supplementary Figure S3). Genome copies were inferred based on NCBI BLAST of 16 s gene copies per organism. qPCR was run in duplicate. Sex-independent p-values reported above relevant groups. LOQ = 5.5445. Comparisons drawn between groups for **A.** ASF 356, **B.** ASF 360, **C.** ASF 361, **D.** ASF 457, **E.** ASF 492, **F.** ASF 500, **G.** ASF 502, and **H.** ASF 519. *IL10*, interleukin 10. *n* = 3 WT mice per males and females, respectively, per group at all timepoints excepting *n* = 2 WT non-inoculated males at 3-DPI. *n* = 3 *IL10^−/−^* mice per males and females, respectively, per group at 3- and 8-DPI excepting *n* = 4 *IL10^−/−^* inoculated females at 8-DPI. *n* = 4 *IL10^−/−^* mice per males and females, respectively, per group at 14-DPI.*, *p*-value < 0.05; **, *p*-value < 0.01; ***, *p*-value < 0.001; ****, *p*-value < 0.0001.

**Figure 14:**
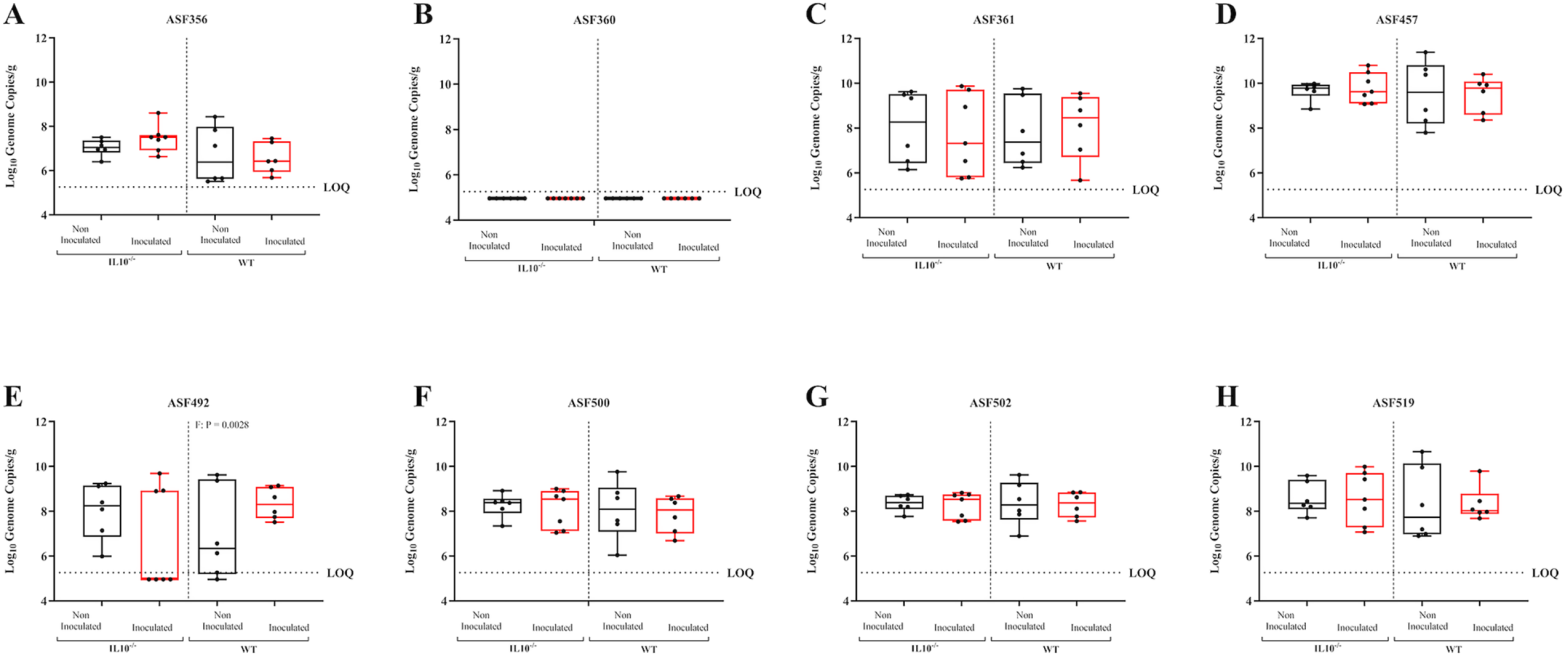
Effect of SPtA inoculation on resident microbiota 8-DPI. Log10 Genome Copies of each ASF member per gram ceca tissue extracted from detected in 129S6/SvEv WT and 129S6/SvEv *IL10^−/−^* genetic background either inoculated with *S.* paratyphi or PBS at 3 DPI. Ct values for each primer pair were fit to a linear regression generated using dilutions of cloning vectors containing the 16 s rRNA gene of each member (Supplementary Figure S3). Genome copies were inferred based on NCBI BLAST of 16 s gene copies per organism. qPCR was run in duplicate. Sex-independent p-values reported above relevant groups. LOQ = 5.2622. Comparisons drawn between groups for **A.** ASF 356, **B.** ASF 360, **C.** ASF 361, **D.** ASF 457, **E.** ASF 492, **F.** ASF 500, **G.** ASF 502, and **H.** ASF 519. *IL10*, interleukin 10. *n* = 3 WT mice per males and females, respectively, per group at all timepoints excepting *n* = 2 WT non-inoculated males at 3-DPI. *n* = 3 *IL10^−/−^* mice per males and females, respectively, per group at 3- and 8-DPI excepting *n* = 4 *IL10^−/−^* inoculated females at 8-DPI. *n* = 4 *IL10^−/−^* mice per males and females, respectively, per group at 14-DPI. *, *p*-value < 0.05; **, *p*-value < 0.01; ***, *p*-value < 0.001; ****, *p*-value < 0.0001.

**Figure 15:**
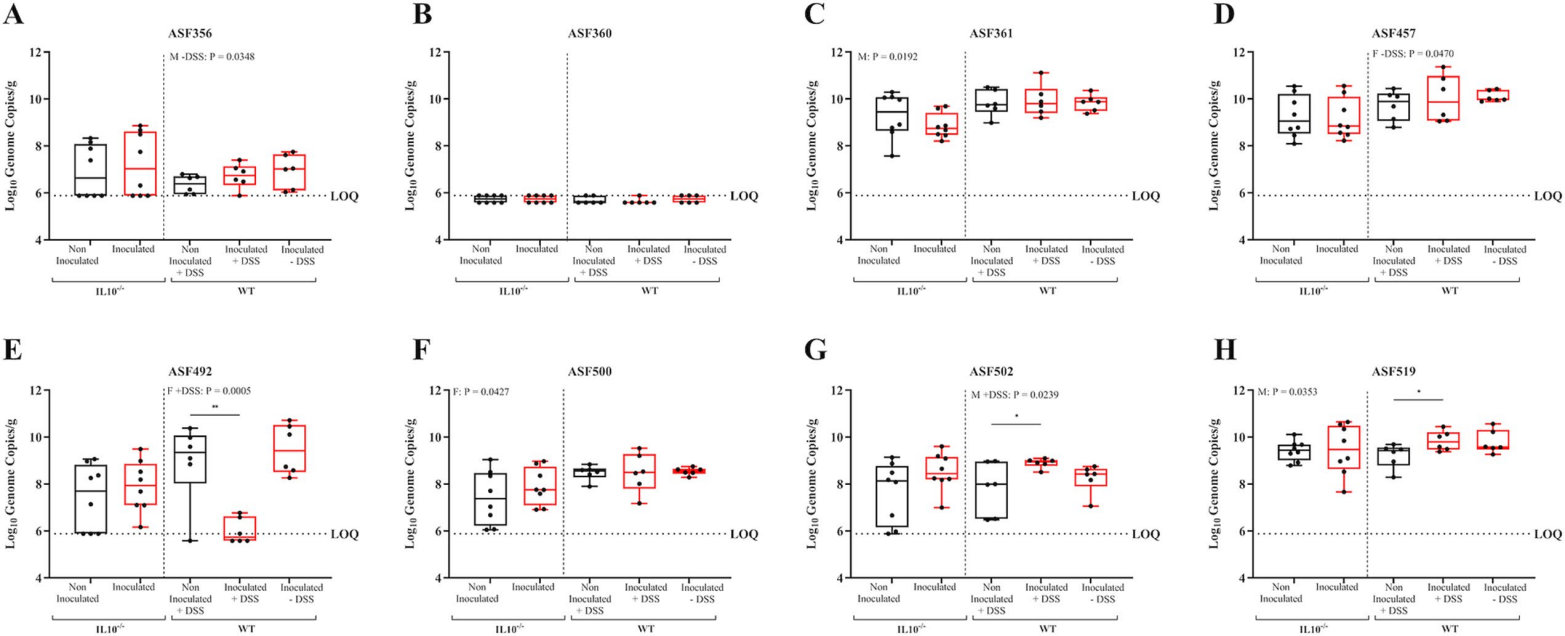
Effect of SPtA inoculation on resident microbiota 14-DPI. Log10 Genome Copies of each ASF member per gram ceca tissue extracted from detected in 129S6/SvEv WT and 129S6/SvEv *IL10^−/−^* genetic background either treated with 2% DSS (WT only) or PBS and inoculated with *S.* paratyphi or PBS. Ct values for each primer pair were fit to a linear regression generated using dilutions of cloning vectors containing the 16 s rRNA gene of each member (Supplementary Figure S3). Genome copies were inferred based on NCBI BLAST of 16 s gene copies per organism. qPCR was run in duplicate. LOQ = 5.8862. Comparisons drawn between groups for **A.** ASF 356, **B.** ASF 360, **C.** ASF 361, **D.** ASF 457, **E.** ASF 492, **F.** ASF 500, **G.** ASF 502, and **H.** ASF 519. *IL10*, interleukin 10. *n* = 3 WT mice per males and females, respectively, per group at all timepoints excepting *n* = 2 WT non-inoculated males at 3-DPI. *n* = 3 *IL10^−/−^* mice per males and females, respectively, per group at 3- and 8-DPI excepting *n* = 4 *IL10^−/−^* inoculated females at 8-DPI. *n* = 4 *IL10^−/−^* mice per males and females, respectively, per group at 14-DPI. Sex-independent p-values reported above relevant groups. *IL10*, interleukin 10; *, *p*-value < 0.05; **, *p*-value < 0.01; ***, *p*-value < 0.001; ****, *p*-value < 0.0001.

All statistics were accomplished utilizing GraphPad Prism version 10. The results described in Figures 1–5 were analyzed utilizing a two-way analysis of variance (two-way ANOVA) full model assuming sphericity with Sidak’s correction for multiple comparisons and single pooled variance. The results described in Figures 6–8 were analyzed using a two-way ANOVA with Tukey’s correction for multiple comparisons and single pooled variance. The results presented in Figures 9, 10 were analyzed using unpaired *t*-tests on log-transformed 2_-ΔΔct_ values with an unpaired Gaussian distribution assuming a parametric distribution and same standard deviation with Holm-Sidak’s correction for multiple comparisons. The results presented in Figures 11, 12 were analyzed with a one-way ANOVA, no matching, Gaussian distribution, assuming equal standard deviations, and Sidak’s correction for multiple comparisons with a single pooled variance. The results presented in Figures 13–15 were analyzed using unpaired *t*-tests.

## 3 Results

### 3.1 Change in weight is different between inflammatory models

To determine the effect of SPtA infection on host weight change, the weight of mice was measured at 2- to 3-day intervals and recorded as a percentage of initial weight at 3-day post-inoculation (DPI) (Figure 1 and Supplementary Figure S1). Weight gain significance and comparisons are summarized in Supplementary Figure S1. In the wild-type (WT) group, 129S6/SvEv SPtA-inoculated mice showed a significant decrease in weight than the non-inoculated mice at 0- (*p <* 0.05), 7- (*p <* 0.01), 10- (*p <* 0.05), and 12-DPI (*p <* 0.0001) for males and at 7- (*p <* 0.05) and 12-DPI (*p <* 0.001) for females (Figures 1C,H). In the dextran sulfate sodium (DSS)-treated group, 129S6/SvEv inoculated females showed a significant (*p <* 0.05) increase in weight compared to 129S6/SvEv non-inoculated females at 12-DPI (Figure 1I). In the chronic inflammatory 129S6/SvEv *IL10^−/−^* group, 129S6/SvEv *IL10^−/−^* inoculated males had a significant decrease in weight at 7- (*p <* 0.001) and 12-DPI (*p <* 0.05) compared to 129S6/SvEv *IL10^−/−^* non-inoculated males (Figure 1E). No significant differences were observed in weight gain between inoculated and non-inoculated mice in the C3H/HeN and 129S6/SvEv male DSS groups.

Comparing between inflammatory models, non-DSS-treated C3H/HeN males showed a significant increase in weight compared to DSS-treated C3H/HeN males at 10- (*p <* 0.0001), 12- (*p <* 0.0001), and 14-DPI (*p <* 0.0001) (Figure 2A). Conversely, in DSS-treated 129S6/SvEv females, a significant (*p <* 0.001) increase in weight was observed compared to respective non-DSS-treated females at 12-DPI (Figure 2F).

### 3.2 Fecal shedding of SPtA was detected in ASF mice

To determine host colonization of SPtA throughout the study, fecal shedding was assessed at 2- to 3-day intervals (Supplementary Figure S2). All mice inoculated with SPtA demonstrated an initial fecal shedding of the bacteria at 3-DPI and throughout the study. Additionally, all inoculated mice demonstrated a peak shedding at 10-DPI, averaging 5.74 Log_10_ (CFU/g), except 129S6/SvEv *IL10^−/−^* male mice, which reached a peak of shedding of 5.83 Log_10_ (CFU/g) by 12-DPI (Figure 3). All non-inoculated control mice tested negative for SPtA fecal shedding at any time tested.

In the WT group, 129S6/SvEv males had significantly (*p <* 0.05) greater SPtA shedding than 129S6/SvEv females at 7-DPI (Figure 3B); however, in C3H/HeN mice, no significant differences were observed between males and females at any time tested. Comparing between genetic backgrounds (Figure 4), 129S6/SvEv males had significantly greater SPtA shedding than C3H/HeN males at 5- (*p <* 0.01), 7- (*p <* 0.05), and 12-DPI (*p <* 0.05) (Figure 4A).

In the DSS-treated group, C3H/HeN females had significantly greater SPtA fecal shedding than C3H/HeN males at 3- (*p <* 0.0001), 5- (*p <* 0.0001), and 7-DPI (*p <* 0.05) (Figure 3D). The inverse trend in sexes was observed in the 129S6/SvEv mice, as 129S6/SvEv males had significantly greater SPtA shedding than 129S6/SvEv females at 10- (*p <* 0.001) and 12-DPI (*p <* 0.05) (Figure 3E). However, in 129S6/SvEv mice, males had significantly (*p <* 0.001) greater SPtA shedding than C3H/HeN males at 3-DPI (Figure 4B). The inverse significant difference between genetics was observed in C3H/HeN females, as they showed significantly greater SPtA fecal shedding than 129S6/SvEv females at 5- (*p <* 0.001), 7- (*p <* 0.0001), 10- (*p <* 0.0001), and 12-DPI (*p <* 0.001) (Figure 4F).

Compared to the non-treated WT group, the DSS-treated C3H/HeN males had significantly (*p <* 0.001) greater SPtA fecal shedding than the non-DSS-treated C3H/HeN males at 3-DPI (Figure 5A). The DSS-treated C3H/HeN females also showed significantly (*p <* 0.05) greater SPtA fecal shedding than the non-DSS-treated C3H/HeN females at 5-DPI (Figure 5E). Conversely, non-DSS-treated 129S6/SvEv males showed significantly greater SPtA shedding than DSS-treated 129S6/SvEv males at 5- (*p <* 0.01), 7- (*p <* 0.01), and 12-DPI (*p <* 0.05) (Figure 5B).

In the chronic inflammatory 129S6/SvEv *IL10^−/−^* group, differences were observed between sexes and genetics. 129S6/SvEv *IL10^−/−^* females demonstrated significantly greater SPtA shedding than 129S6/SvEv *IL10^−/−^* males at 3- (*p <* 0.05) and 5-DPI (*p <* 0.05) (Figure 3C). Compared to the other groups, 129S6/SvEv *IL10^−/−^* females had significantly greater SPtA fecal shedding than 129S6/SvEv WT females at 3- (*p <* 0.05) and 7-DPI (*p <* 0.001) (Figure 5G). This same difference was observed between 129S6/SvEv *IL10^−/−^* females and DSS-treated 129S6/SvEv WT females, with greater fecal shedding observed at 3- (*p <* 0.01), 5- (*p <* 0.001), 7- (0.0001), 10- (*p <* 0.0001), and 12-DPI (*p <* 0.0001) (Figure 5H). 129S6/SvEv *IL10^−/−^* females also demonstrated significantly (*p* < 0.05) greater SPtA fecal shedding than non-DSS-treated C3H/HeN females at 12-DPI (Figure 4G). Conversely, 129S6/SvEv *IL10^−/−^* females demonstrated significantly (*p* < 0.05) lower SPtA fecal shedding than DSS-treated C3H/HeN females at 5- and 10-DPI (Figure 4H).

### 3.3 Sex and inflammation affected susceptibility to tissue colonization and invasion

To assess extraintestinal invasion of SPtA, liver and spleen were collected and screened for SPtA at necropsy. SPtA colonization/invasion significance and comparisons are summarized in Supplementary Figure S3. In the WT group, at 3-DPI, ~33% (two females) of 129S6/SvEv mice showed colonization of the spleen, and ~17% (one female) of 129S6/SvEv mice showed colonization of the liver. At 8 DPI, ~33% (one female and one male) of 129S6/SvEv mice showed colonization of the spleen, and ~17% (one male) of 129S6/SvEv mice showed colonization of the liver. At 14 DPI, ~33% (one female) of C3H/HeN mice showed no colonization of the ceca content, 50% (two female and one male) of C3H/HeN mice showed no colonization of the ileal content, and ~17% (one female) of C3H/HeN mice showed colonization of the spleen. No significant differences in colonization between non-DSS-treated C3H/HeN mice were observed. In the DSS-treated group, at 14-DPI, ~17% (one female) of 129S6/SvEv mice showed no colonization of any intestinal content, ~33% (two males) of C3H/HeN and ~17% (one female) of 129S6/SvEv mice showed no colonization of the spleen, and ~33% (one female and one male) of C3H/HeN mice showed colonization of the liver. 129S6/SvEv females have significantly (*p* < 0.05) higher colonization of the colon content than 129S6/SvEv males at 3 DPI (Figure 8C). 129S6/SvEv males had significantly higher colonization of ceca (*p* < 0.01) and colon (*p* < 0.01) content than 129S6/SvEv females at 14 DPI (Figures 8L,M). The effect of DSS-treatment on colonization and invasion at 3- and 8-DPI was not tested.

In the chronic inflammatory 129S6/SvEv *IL10^−/−^* group, at 3-DPI, ~83% (five females) of 129S6/SvEv *IL10^−/−^* mice showed colonization of the spleen, and 50% (three females) of 129S6/SvEv *IL10^−/−^* mice showed colonization of the liver. At 8 DPI, ~14% (one female) 129S6/SvEv *IL10^−/−^* mice showed colonization of the spleen, and ~29% (one male and one female) of 129S6/SvEv *IL10^−/−^* mice showed colonization of the liver. At 14 DPI, 12.5% (one male) of 129S6/SvEv *IL10^−/−^* mice showed no colonization of the ceca and ileal content, ~37.5% (three females) of 129S6/SvEv *IL10^−/−^* mice showed colonization of the spleen, and 50% (four females) of 129S6/SvEv *IL10^−/−^* mice showed colonization of the liver. 129S6/SvEv *IL10^−/−^* female ilea content showed significantly (*p <* 0.001) greater detection than 129S6/SvEv males (Figure 6A). Additionally, 129S6/SvEv *IL10^−/−^* female spleen tissue showed significantly (*p <* 0.001) greater detection than 129S6/SvEv males (Figure 6D). 129S6/SvEv *IL10^−/−^* female liver tissue also showed significantly (*p <* 0.0001) greater detection than 129S6/SvEv males (Figure 6E).

Comparing between groups, 129S6/SvEv *IL10^−/−^* showed greater susceptibility to colonization and invasion than other genetic backgrounds. At 3 DPI, 129S6/SvEv *IL10*^−/−^ males demonstrated significantly (*p* < 0.05) greater colonization of the spleen (Figure 8D). At 14 DPI, 129S6/SvEv *IL10^−/−^* female ilea content showed significantly greater colonization than 129S6/SvEv WT (*p <* 0.05) and C3H/HeN (*p <* 0.001) female mice (Figure 6A). 129S6/SvEv *IL10^−/−^* female colon content also showed significantly (*p <* 0.05) greater colonization than C3H/HeN females (Figure 6C). 129S6/SvEv *IL10^−/−^* female spleen tissue showed significantly greater detection than respective 129S6/SvEv WT (*p <* 0.01) and C3H/HeN (*p <* 0.05) female mice (Figure 6D). 129S6/SvEv *IL10^−/−^* female liver tissue also showed significantly greater detection than respective 129S6/SvEv WT (*p <* 0.0001) and C3H/HeN females (*p <* 0.0001) (Figure 6E).

### 3.4 Inflammation affects susceptibility to tissue colonization and invasion

To further assess the effect of inflammation on the ability of SPtA to colonize the GI tract of tested mice and invade extraintestinal tissues, comparisons of SPtA colonization of both were drawn (Supplementary Figure S3). In the WT non-treated group, no significant differences were detected among the mice tested. At 14 DPI, DSS-treated C3H/HeN mice had significantly greater colonization of ceca content (*p <* 0.01) and colon content (*p <* 0.01) than respective DSS-treated 129S6/SvEv mice (Figures 7B,C). Additionally, it was observed that non-DSS-treated 129S6/SvEv mice had significantly (*p* < 0.05) greater colonization of the colon content than non-DSS-treated C3H/HeN mice (Figure 7C).

In the chronic inflammatory 129S66/SvEv *IL10^−/−^* group, at 8 DPI, 129S6/SvEv *IL10^−/−^* female colon content had significantly (*p <* 0.01) lower detection of SPtA than 129S6/SvEv *IL10^−/−^* male mice (Figure 8H). At 14 DPI, 129S6/SvEv *IL10^−/−^* female ileal content had significantly (*p <* 0.01) greater SPtA detection than 129S6/SvEv *IL10^−/−^* males (Figure 8K). 129S6/SvEv *IL10^−/−^* female spleen tissue showed significantly (*p <* 0.001) greater SPtA detection than 129S6/SvEv *IL10^−/−^* males (Figure 8N). 129S6/SvEv *IL10^−/−^* female also showed significantly (*p <* 0.0001) greater SPtA detection in liver tissues than 129S6/SvEv *IL10^−/−^* males (Figure 8O).

When comparing between inflammatory groups, at 3 DPI, 129S6/SvEv *IL10^−/−^* males had significantly (*p <* 0.05) higher levels of SPtA detected in colonic contents than 129S6/SvEv WT males (Figure 8C). At 8 DPI, 129S6/SvEv *IL10^−/−^* female mice showed significantly (*p <* 0.01) lower colonic colonization than respective 129S6/SvEv WT females (Figure 8H). At 14 DPI, 129S6/SvEv *IL10^−/−^* female intestinal content had significantly greater colonization than DSS-treated 129S6/SvEv WT female mice in the ilea, ceca, and colon (*p <* 0.001, *p <* 0.01, *p <* 0.001) and non-DSS-treated 129S6/SvEv WT female mice in the ilea (*p <* 0.05) (Figures 8K,L,M). DSS-129S6/SvEv female colon content also had significantly (*p <* 0.05) lower colonization than non-DSS-treated 129S6/SvEv WT females (Figure 8M). 129S6/SvEv *IL10^−/−^* female spleen and liver tissue showed significantly greater detection than both DSS-treated 129S6/SvEv WT females (*p <* 0.05, *p <* 0.0001) and non-DSS-treated 129S6/SvEv WT females (*p <* 0.01, *p <* 0.0001) (Figures 8N,O). At 14 DPI, DSS-treated C3H/HeN mice (pooled sex) showed significantly greater colonization of ilea (*p* < 0.05), ceca (*p <* 0.01), and colon content (*p <* 0.01) than respective non-DSS-treated C3H/HeN mice (Figures 7A–C). The inverse was observed in the 129S6/SvEv mice colon content, where non-DSS-treated mice showed significantly (*p* < 0.05) greater colonization than DSS-treated 129S6/SvEv mice (Figure 7C).

### 3.5 SPtA infection affected tight junction protein expression

To assess the effect of SPtA on the integrity of the intestinal epithelia, tight junction proteins in the ceca were assessed using qPCR and cecal cDNA (Figure 9 and Supplementary Figure S4).

In the WT group, there were no significant differences in any of the tight junction proteins tested at any time point (Figures 9A–C). However, at 3 DPI, 129S6/SvEv WT mice (sex combined) had numerically greater expression of *ZO-1* than 129S6/SvEv WT non-inoculated mice (Figure 9A).

In the chronic inflammatory 129S6/SvEv *IL10^−/−^* group, inoculated mice (sex combined) had numerically lower expression of Occludin than 129S6/SvEv *IL10^−/−^* non-inoculated mice at 8 DPI (Figure 9E). At 14 DPI, 129S6/SvEv *IL10^−/−^* inoculated females had significantly greater expression of *Ocln* and *ZO-1* (*p <* 0.05) than 129S6/SvEv *IL10^−/−^* non*-*inoculated females (Figure 9F).

### 3.6 SPtA inoculation reduced pro-inflammatory cytokine expression

To assess the impact of SPtA inoculation on cytokine expression in the ileum, we conducted quantitative PCR on reverse-transcribed ileal RNA (Figure 10). In the WT group, at 3 DPI, 129S6/SvEv inoculated females showed significantly (*p <* 0.05) lower expression of *IL6* than 129S6/SvEv WT non*-*inoculated females (Figure 10A). No significant differences were observed between mice treated with DSS or the chronic inflammatory *IL10^−/−^* group (Figures 10C–F).

### 3.7 Inflammatory conditions and length of SPtA infection affected intestinal pathology

To assess the effect of SPtA colonization on the pathology of the ceca, harvested and fixed ceca tissues from all mice were analyzed by a certified pathologist and assigned scores associated with epithelial integrity, submucosal edema, and polymorphonuclear leukocyte infiltration as described previously (Figure 11 and Supplementary Figure S5) (Suar et al., 2006).

No significant differences were observed when comparing within any of the groups tested.

Comparison between groups shows that at 14 DPI, significantly (*p <* 0.05) greater pathology was observed in 129S6/SvEv *IL10^−/−^* inoculated males than non-DSS-treated 129S6/SvEv WT inoculated males (Figure 11C).

However, drawing statistical comparisons between identical mouse groups at different timepoints showed differences in the progression of pathology over time (Figure 12).

In the WT group, both inoculated and non-inoculated 129S6/SvEv males as well as non-inoculated 129S6/SvEv females showed significantly (*p <* 0.05) greater pathology at 14 DPI compared to 8 DPI 129S6/SvEv non-inoculated mice of the respective sex (Figures 12C,D).

No differences were observed between non-DSS- and DSS-treated mice.

In the chronic inflammatory 129S6/SvEv *IL10*^−/−^ group, both non-inoculated (*p <* 0.05) and inoculated (*p <* 0.01) males had significantly greater pathology at 14 DPI than the same groups at 3- and 8-DPI (Figures 12A,B).

### 3.8 SPtA colonization affected mice microbiota

To assess the effect of SPtA on the resident microbiota in mice, the levels of each of the eight ASF taxa within the ceca contents of all mice were enumerated by qPCR following necropsy (Figures 13–15; and Supplementary Figure S6). Resident microbes were detected in all mice, as noted in the figures, with mice having too few gene copies of a specific taxon to detect in any particular assay being marked as one-half of the detection limit determined for each timepoint by the sample with the highest mass. This was particularly prevalent with ASF 360, as this taxon was undetected in the majority of mice screened.

In the WT group, 129S6/SvEv inoculated females showed significantly lower detection of ASF 500 (*p <* 0.05) and ASF 502 (*p* < 0.04) than 129S6/SvEv non-inoculated females at 3 DPI (Figure 13F). At 8 DPI, 129S6/SvEv inoculated females showed significantly (*p* < 0.01) greater detection of ASF 492 than 129S6/SvEv non-inoculated females (Figure 14E). At 14 DPI, 129S6/SvEv inoculated males showed significantly greater detection of ASF 356 (*p <* 0.05) than 129S6/SvEv non-inoculated males (Figure 15A). Additionally, 129S6/SvEv inoculated females showed significantly (*p <* 0.05) greater detection of ASF 457 than 129S6/SvEv non-inoculated females at 14 DPI (Figure 15D).

In the DSS-treated group at 14 DPI, 129S6/SvEv inoculated mice (sex combined) showed significantly lower detection of ASF 492 (*p* < 0.01) and greater detection of ASF 502 (*p* < 0.05) and ASF 519 (*p* < 0.05) than non-inoculated 129S6/SvEv mice (Figures 15E,G,H). 129S6/SvEv inoculated male also showed significantly (*p <* 0.05) greater detection of ASF 502 than 129S6/SvEv non-inoculated males (Figure 15G). Additionally, 129S6/SvEv inoculated females showed significantly lower detection of ASF 492 (*p <* 0.001) than 129S6/SvEv non-inoculated females (Figure 15E). The effects of DSS-treatment on microbial composition at 3- and 8-DPI were not tested.

In the chronic inflammatory 129S6/SvEv *IL10^−/−^* group, at 3 DPI, inoculated females showed significantly greater detection in the ceca of ASF 356 (*p <* 0.05), ASF 492 (*p <* 0.01), and ASF 500 (*p <* 0.01) than non-inoculated females (Figures 13A,E,F). Inoculated males showed significantly (*p <* 0.05) lower detection in the ceca of ASF 457 than non-inoculated males (Figure 13D). At 14 DPI, inoculated females showed significantly (*p <* 0.05) greater detection in the ceca of ASF 500 than 129S6/SvEv *IL10^−/−^* non-inoculated females (Figure 15F). Additionally, 129S6/SvEv *IL10^−/−^* inoculated males showed significantly lower detection of ASF 361 (*p <* 0.05) and greater detection of ASF 519 (*p* < 0.05) than 129S6/SvEv *IL10^−/−^* non-inoculated males (Figures 15C,H). No significant changes were detected at 8 DPI within the 129S6/SvEv *IL10^−/−^* group (Figure 14).

## 4 Discussion

Paratyphoid fever is an emergent systemic human disease that is endemic in many Southeast Asian countries. This disease is critically under-researched, partly due to the lack of a sufficient model to study the disease (Hormaeche, 1979b, 1979a; Simon et al., 2011; Furuse, 2019). Few experimental models have been generated displaying systemic typhoidal infection in mice, although none are sufficiently representative of humans (Palmer and Slauch, 2017). Previously, streptomycin pre-treated mouse models were shown to serve as a poor model due to a deficient microbiota inherent to a developed enteric immune response and the inability to effectively and efficiently monitor whole microbiota fluctuations induced by SPtA colonization due to either depletion or complex microbiome composition (Suar et al., 2006; Javier Santander and Curtiss, 2010; Dobinson et al., 2017).

We have previously demonstrated that mice harboring the ASF as a sufficient, limited microbiota model to allow for the colonization of *Enterobacteriaceae* while also retaining select benefits of a full microbiota not present in germ-free models (Wymore Brand et al., 2015). C3H/HeN mice serve as a good high-stress temporal inflammation model when treated with 2% DSS, as they are known to have an elevated inflammatory response to treatment (Mähler et al., 1998). 129S6/SvEv *IL10^−/−^*mice were made available as a chronic inflammation model (Rennick and Fort, 2000; Keubler et al., 2015; Overstreet et al., 2021). 129S6/SvEv WT mice served as a control for the *IL10^−/−^* mice, as well as an additional DSS-induced temporal inflammation model compared to C3H/HeN mice.

Following oral ingestion of typhoidal *Salmonella*, the disease progresses by establishing intestinal colonization, which, along with the subsequent expansion to extraintestinal tissues, allows bacteria to replicate and shed through the host feces (Johnson et al., 2018; Gibani et al., 2019). Fecal shedding of *Salmonella*, including SPtA, is an important indicator of colonization, particularly when sustained over an extended period after infection, as it signifies active replication of the bacteria in the intestinal tracts of the host organism (Murase et al., 2000; Chong et al., 2021). In this study, we observed trends in shedding over the 15-day period, indicating that the most readily colonized group was 129S6/SvEv *IL10^−/−^* females (Figures 3–5). 129S6/SvEv *IL10^−/−^* mice trended toward greater shedding than the next highest observed group, DSS-treated C3H/HeN mice (Figures 3–5). Prior studies consistently noted that C3H/HeN mice are highly susceptible to DSS, which correlates with fecal shedding patterns more closely resembling those of the chronically inflamed 129S6/SvEv *IL10^−/−^* model. This is attributed to DSS’s impact on intestinal mucus production and tight junction barrier function, which in turn increases susceptibility to pathogen colonization (Mähler et al., 1998; Suzuki et al., 2005; Yang and Merlin, 2024). In this study, we observed both fecal shedding of SPtA and colonization of multiple regions of the gastrointestinal tract. This is unique in comparison with prior murine models of SPtA infection, as the previous streptomycin-pretreated mouse model only overlapped this study in its observation of ceca content and liver and spleen tissue at 3 DPI (Suar et al., 2006). In a more recent study observing a human model, however, the presence of stool shedding was observed at an amount starting at 1 DPI for less than half of the patients tested, albeit a far lower inoculation dose of 10^3^ CFU (Dobinson et al., 2017).

One of the hallmarks of typhoidal *Salmonella* infection, separating it from other pathogenic *Salmonella* strains, is the subsequent enteric fever that follows initial infection (Xie et al., 2022). This infection is established through intestinal invasion of the microfold cells of Peyer’s patches, where it spreads through the lymphatic vessels and replicates in the phagocytes of the spleen and liver (Dobinson et al., 2017; Manesh et al., 2021; Xie et al., 2022). Splenomegaly investigated in this study at 14 DPI showed no significant changes in spleen mass between groups (Supplementary Figure S8). Significantly (*p* > 0.05) higher shedding, as well as the observations of extraintestinal colonization of the liver and spleen, were observed in 129S6/SvEv *IL10^−/−^* female mice over all other models tested (Figures 3–6, 8–10). This sex-tied relationship is consistent with prior observations of 129S6/SvEv *IL10^−/−^* mice, where female mice have demonstrated a higher propensity toward intestinal inflammation and dysbiosis, and it is posited that inflammation is a key component of the progression of *Salmonella* pathogenesis (Barman et al., 2008; Ferreira et al., 2011; Schultz et al., 2018; Casado-Bedmar et al., 2023). Of note, SPtA is less virulent in host humans than other typhoidal *Salmonella* in part due to the absence of a Vi capsule and the presence of various pseudogenes in place of virulence factors (Dobinson et al., 2017; Johnson et al., 2018). As such, this serves as a preferable model of infection to that seen in the Typhimurium model, where disease mirrors that of ST (Dobinson et al., 2017; Johnson et al., 2018).

The results of the current study, as they pertain to colonization, are comparable at 3 DPI to those of the streptomycin pretreated model in the detection of SPtA in the ceca content (Suar et al., 2006). Although extraintestinal invasion of liver and spleen was much lower at 3 DPI in the current study, it can be argued that this is representative of a more robust host model due to the need for a progression of disease that may more accurately mirror that of human infection (Suar et al., 2006; Manesh et al., 2021). Comparison of colonization and invasion results is impossible to make with the controlled human study due to the lack of a ‘necropsy’ component, although it is of note that no mice tested in the present study were assessed for signs of fever or lethargy, as noted in infected human patients (Dobinson et al., 2017).

Intraperitoneal and neonatal methods previously employed for typhoidal *Salmonellae* suffer from inherent limitations such as difficulty in monitoring microbial composition effects, either due to a bypassing of the gastrointestinal colonization route or the presence of a complex host microbiome (Carter and Collins, 1974; Pasetti et al., 2003; Javier Santander and Curtiss, 2010). Additionally, these studies do not investigate changes in host-associated gastrointestinal gene expression induced by SPtA and the pathological changes conferred to intestinal tissues (Carter and Collins, 1974; Pasetti et al., 2003; Javier Santander and Curtiss, 2010). The current study investigated the changes in gene expression of a select set of host genes important in host response to infection across 129S6/SvEv models tested.

An observed increase in the expression of tight junction proteins appears incongruent with expectations of decreased tight junction effectiveness due to the inflammation triggered by SPtA. Mylona et al. previously reported that the long O-polysaccharide chains of SPtA have an effect in decreasing inflammatory responses in hosts (Figure 9) (Mylona et al., 2021). The increase in tight junction protein expression is in contrast to expectations based on previous findings, where proinflammatory cytokines, such as TNF and IL-1β, are seen to inhibit transcription of junction proteins (Figure 9) (Edelblum and Turner, 2009). However, a heightened gasdermin-D-mediated pyroptosis has been previously described. The pyroptotic inflammation pathway, stimulated by SPtA, is initiated by assembling an inflammasome and the expression of pyrin (Yu et al., 2021). An indicator gene of pyroptotic inflammation encoding the pyrin gene, Mediterranean fever virus gene (*MEFV*), was also investigated by qPCR, although no significant differences in expression were observed at any time point (Supplementary Figure S9) (Sharma et al., 2020). Pyrin is found to have inherent anti-tumor effects, among which include the bolstering of tight junctions (Sharma et al., 2018).

Significantly (*p* < 0.05) greater expression of *Ocln* and *ZO-1* was also observed in female inoculated 129S6/SvEv *IL10^−/−^* mice (Figure 9F). Microbe-associated molecular pattern (MAMP) stimulation of TLR2 characteristic of *Salmonellae* pathogenesis, in general, has been demonstrated to heighten *ZO-1-*mediated tight junctions through p70S6K stimulation (Cario et al., 2007). During apoptotic stress, ZO-1 internalizes and is re-localized to the cytoplasmic center of cells, diminishing tight junctions in a post-transcriptional manner that may not be observable through qPCR (Cario et al., 2007).

This study determined significant reductions in pro-inflammatory cytokine *IL6* expression at 3 DPI in the SPtA-inoculated 129S6/SvEv WT and *IL10*^−/−^ mice (Figure 10). These changes correlate well with observed lower pathology at 3 DPI within the same groups (Figures 11, 12). The inflammation inhibition by SPtA is considered a hallmark of the virulence of this bacterium and was previously correlated with the very long O-antigen chains SPtA (Mylona et al., 2021). These data indicate that our model can recapitulate the pathology associated with human infection.

This study also observed a maximal fecal shedding at 14 DPI paired with a decrease in pathology observed through qPCR of tight junction proteins and cytokines, as well as direct pathological scoring, and no lesion pathology. When accounting for fecal shedding and both intestinal and extraintestinal colonization dynamics observed in this study, this model recapitulates a shedding phenotype observed in human infections with both ST and SPtA (Dobinson et al., 2017; Gibani et al., 2018, 2019; Johnson et al., 2018).

Significant increases in pathology at 14 DPI in all mouse groups tested were observed and are congruous with expectations of both chronic 129S6/SvEv *IL10^−/−^* and temporal DSS pro-inflammatory models (Figures 11, 12) (Kühn et al., 1993; Berg et al., 1996; Chassaing et al., 2014; Zurita-Turk et al., 2020). Additionally, the significantly greater total pathology observed between 129S6/SvEv *IL10^−/−^* males and their WT counterparts was expected (Figure 13) (Overstreet et al., 2021). Pathological scoring utilizing the same schematic as that of the streptomycin pre-treated model shows the 129S6/SvEv *IL10^−/−^* model harboring the ASF having greater pathology when carried out to 14 DPI, as expected, although pathology at 3 DPI is lower (Figure 12) (Suar et al., 2006).

Although in this study, observed increases in resident defined-microbiota members are initially incongruous with previously described dysbiotic conditions expected in typhoidal *Salmonella* pathogenesis, the taxonomy of the members clues into possible explanations for such observations (Gillis et al., 2018; Haak et al., 2020). Haak et al. have previously linked infection with typhoidal *Salmonella* to microbial dysbiosis in human observational studies, where microbiota richness and diversity were reduced with decreased anaerobic bacteria and increased potential pathogens (Haak et al., 2020). This study was unable to prevent surveyed patients from receiving antibiotic treatment, and it also lacked control over initial microbiota composition (Haak et al., 2020). In our study, SPtA infection caused increases in several ASF taxa in the 129S6/SvEv *IL10^−/−^* mice, specifically ASF 356, ASF 492, ASF 500, and ASF 519 (Figures 13–15). ASF 356 is classified as *Clostridium* sp., and ASF 500 is classified as *Pseudoflavonifractor* sp., both classified under the order *Clostridiales* (Dewhirst et al., 1999; Sarma-Rupavtarm et al., 2004). A hallmark feature of many *Clostridiales* members is the ability to undergo Stickland metabolism, where the extremely oxygen-sensitive microbes are capable of utilizing environmental amino acids as an end electron acceptor in fermentative metabolism (Pavao et al., 2022). In a pro-inflammatory environment, such as the one stimulated by typhoidal *Salmonella* in humans, higher extracellular access to host amino acids released by pyroptotic cells could confer a metabolic advantage to these *Clostridiales* members (Zhang et al., 2022).

In addition to the 129S6/SvEv *IL10^−/−^* model, we observed heightened density of resident microbes within 129S6/SvEv WT mice. Increases in the population of many of the same taxa were observed, though notably, ASF 492, ASF 500, and ASF 502 were observed as decreased in certain groups of SPtA-inoculated mice at 3- and 14-DPI (Figures 13, 15). We have previously shown that 129S6/SvEv *IL10^−/−^* mice have marked increases in the presence of all ASF members, excluding 457 and 492, when compared to 129S6/SvEv ASF WT mice, possibly indicating the influence of the genetic background and subsequent variation in MHS receptors of mice tested induces fluctuations in resident microbiota (Ott et al., 2020). The only ASF member significantly increased regardless of sex was ASF 519, *Parabacteroides goldsteinii,* at 14 DPI (Figure 15). ASF 519 is often implicated in studies involving ASF mice as the dominant member of the microbiota, possibly explaining consistency in results regardless of sex (Wymore Brand et al., 2015). Additionally, variability of the same ASF taxa across timepoints when compared to the control for the SPtA-inoculated WT 129S6/SvEv ASF mice gave further credence to the superiority of the 129S6/SvEv *IL10^−/−^* mice as a model that mimicked human disease.

This study describes a viable *in vivo* model for oral infection and extraintestinal invasion of *Salmonella enterica subsp. enterica* Serovar Paratyphi A. Mice harboring the altered Schaedler flora defined microbiota demonstrate an open microbial niche that permits SPtA colonization, which is normally closed in conventionally reared mice, as well as a model in which microbial community disruptions is observed. The chronic inflammatory 129S6/SvEv *IL10^−/−^* ASF mice provided the conditions by which intestinal disruption allowed for extraintestinal invasion and the progression of systemic disease. Disruption to the resident ASF microbiota was observed in this infection model, along with modulated transcription of tight junction proteins consistent with leaky gut. The 129S6/SvEv *IL10^−/−^* model also demonstrates an increased pathology of the ceca over time following inoculation. This model serves as a suitable, reproducible, low-risk, and low-investment model for the study of SPtA. Future research into host immune response beyond transcription is needed to validate suitability as a vaccination model.

## Data Availability

The raw data supporting the conclusions of this article will be made available by the authors, without undue reservation.

## Supplementary material

SUPPLEMENTARY FIGURE S1 Summary of significant comparisons in weight gain data. Comparisons drawn in Figure 1 are summarized with arrows pointing from compared group demonstrating lowest mass at a given time point toward group demonstrating highest mass at a given time point. *, *p*-value < 0.05; **, *p*-value < 0.01; ***, *p*-value < 0.001; ****, *p*-value < 0.0001.

SUPPLEMENTARY FIGURE S2 Summary of significant comparisons in fecal shedding data. Comparisons drawn in Figures 2–5 are summarized with arrows pointing from compared group demonstrating lowest fecal shedding of SPtA at a given time point toward group demonstrating highest fecal shedding of SPtA at a given time point. *IL10*, interleukin 10; *, *p*-value < 0.05; **, *p*-value < 0.01; ***, *p*-value < 0.001; ****, *p*-value < 0.0001.

SUPPLEMENTARY FIGURE S3 Summary of significant comparisons in colonization data. Comparisons drawn in Figures 6–8 are summarized with arrows pointing from compared group demonstrating lowest detection of SPtA in intestinal content or a given tissue at a given time point toward group demonstrating highest detection of SPtA in intestinal content or a given tissue at a given time point. *, *p*-value < 0.05; **, *p*-value < 0.01; ***, *p*-value < 0.001; ****, *p*-value < 0.0001.

SUPPLEMENTARY FIGURE S4 Summary of significant comparisons in tight junction protein data. Comparisons drawn in Figure 9 are summarized with arrows pointing from compared group demonstrating lowest expression of a given tight junction protein at a given time point toward group demonstrating highest expression of a given tight junction protein at a given time point. *, *p*-value < 0.05; **, *p*-value < 0.01; ***, *p*-value < 0.001; ****, *p*-value < 0.0001.

SUPPLEMENTARY FIGURE S5 Summary of significant comparisons in pathology data. Comparisons drawn in Figures 11, 12 are summarized with arrows pointing from compared group demonstrating lowest pathological score at a given time point toward group demonstrating highest fecal shedding at a given time point. *, *p*-value < 0.05; **, *p*-value < 0.01; ***, *p*-value < 0.001; ****, *p*-value < 0.0001.

SUPPLEMENTARY FIGURE S6 Summary of significant comparisons in microbiota data. Comparisons drawn in Figures 13–15 are summarized with arrows pointing from compared group demonstrating lowest abundance of a given taxa at a given time point toward group abundance of a given taxa at a given time point. *, *p*-value < 0.05; **, *p*-value < 0.01; ***, *p*-value < 0.001; ****, *p*-value < 0.0001.

SUPPLEMENTARY FIGURE S7 ASF qPCR standard curves. Linear regression fit models of 5-fold dilutions of cloning vectors containing the 16 s rRNA gene of each ASF member detected using qPCR. R^2^ values and linear regression equations are displayed as well as 95% confidence intervals as displayed by the black hashed lines. qPCR was run in triplicate.

SUPPLEMENTARY FIGURE S8 Relative spleen mass of D14 spleen mass. Relative mass of mouse spleen to mouse weight in grams at necropsy of male and female mice of either C3H/HeN or 129S6/SvEv genetic background either treated with 2% DSS or PBS or *IL10^−/−^*. Comparisons drawn based on inflammatory model and sex. *, *p*-value < 0.05; **, *p*-value < 0.01; ***, *p*-value < 0.001; ****, *p*-value < 0.0001.

SUPPLEMENTARY FIGURE S9 Effect of SPtA inoculation on *MEFV* cytokine expression. Relative 2^-ΔΔCT^ expression of *MEFV* in cecal cDNA extracted from non-DSS-treated 129S6/SvEv WT, DSS-treated 129S6/SvEv WT, or 129S6/SvEv *IL10^−/−^* inoculated with *S.* paratyphi or PBS 3-, 8-, or 14-DPI. qPCR was run in duplicate. *MEFV,* Mediterranean fever virus gene. *, *p*-value < 0.05; **, *p*-value < 0.01; ***, *p*-value < 0.001; ****, *p*-value < 0.0001.
