# Supplementary figures and images for: A defined microbiota mouse model for Salmonella Paratyphi A oral infection
Source: Front Microbiol. 2026 May 19;17:1824783. doi: 10.3389/fmicb.2026.1824783 (PMC13226583; doi:10.3389/fmicb.2026.1824783)

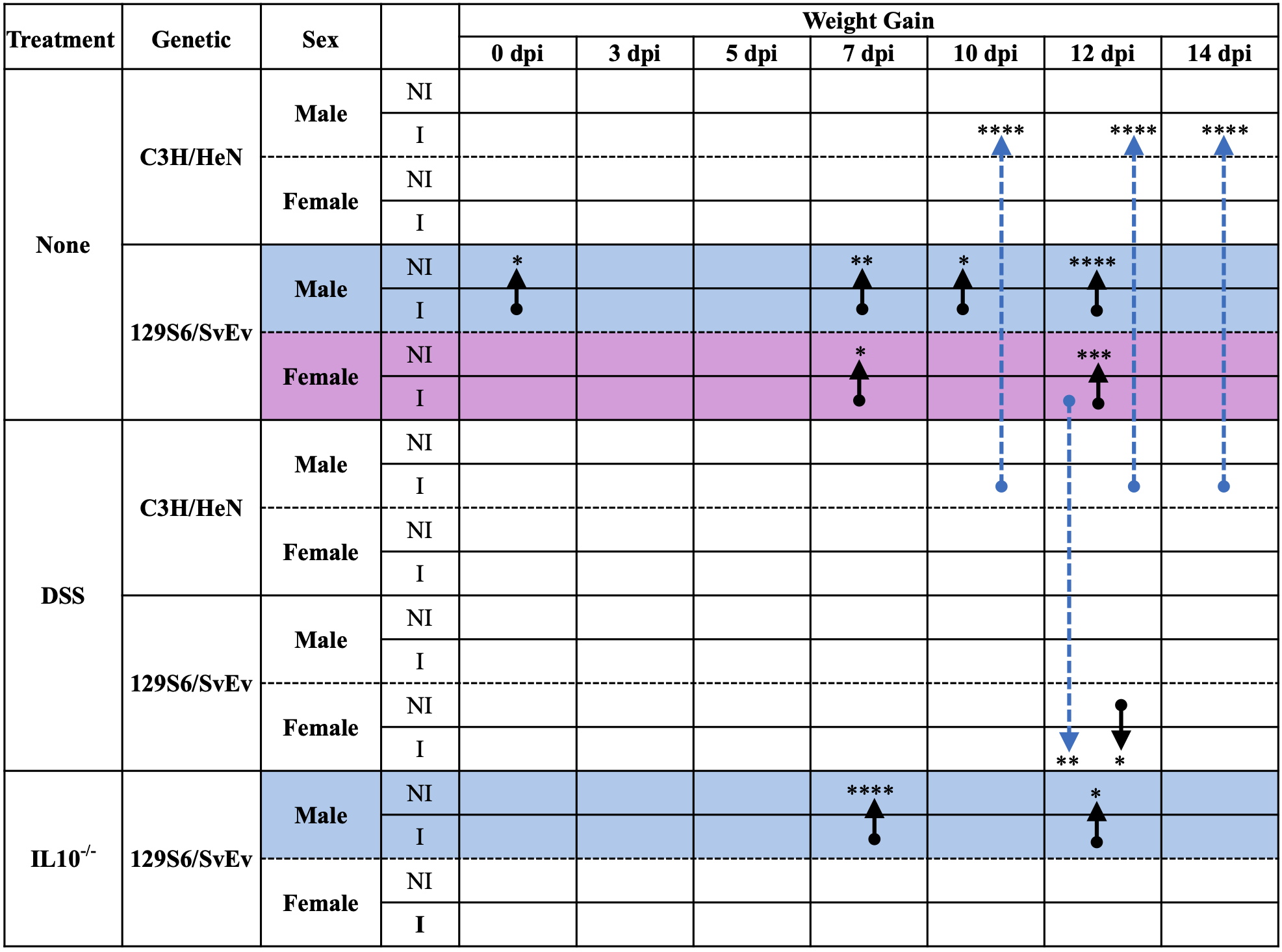

Supplement: SUPPLEMENTARY FIGURE S1 — Summary of significant comparisons in weight gain data. Comparisons drawn in Figure 1 are summarized with arrows pointing from compared group demonstrating lowest mass at a given time point toward group demonstrating highest mass at a given time point. *, p-value < 0.05; **, p-value < 0.01; ***, p-value < 0.001; ****, p-value < 0.0001. [file Image_1.tiff]

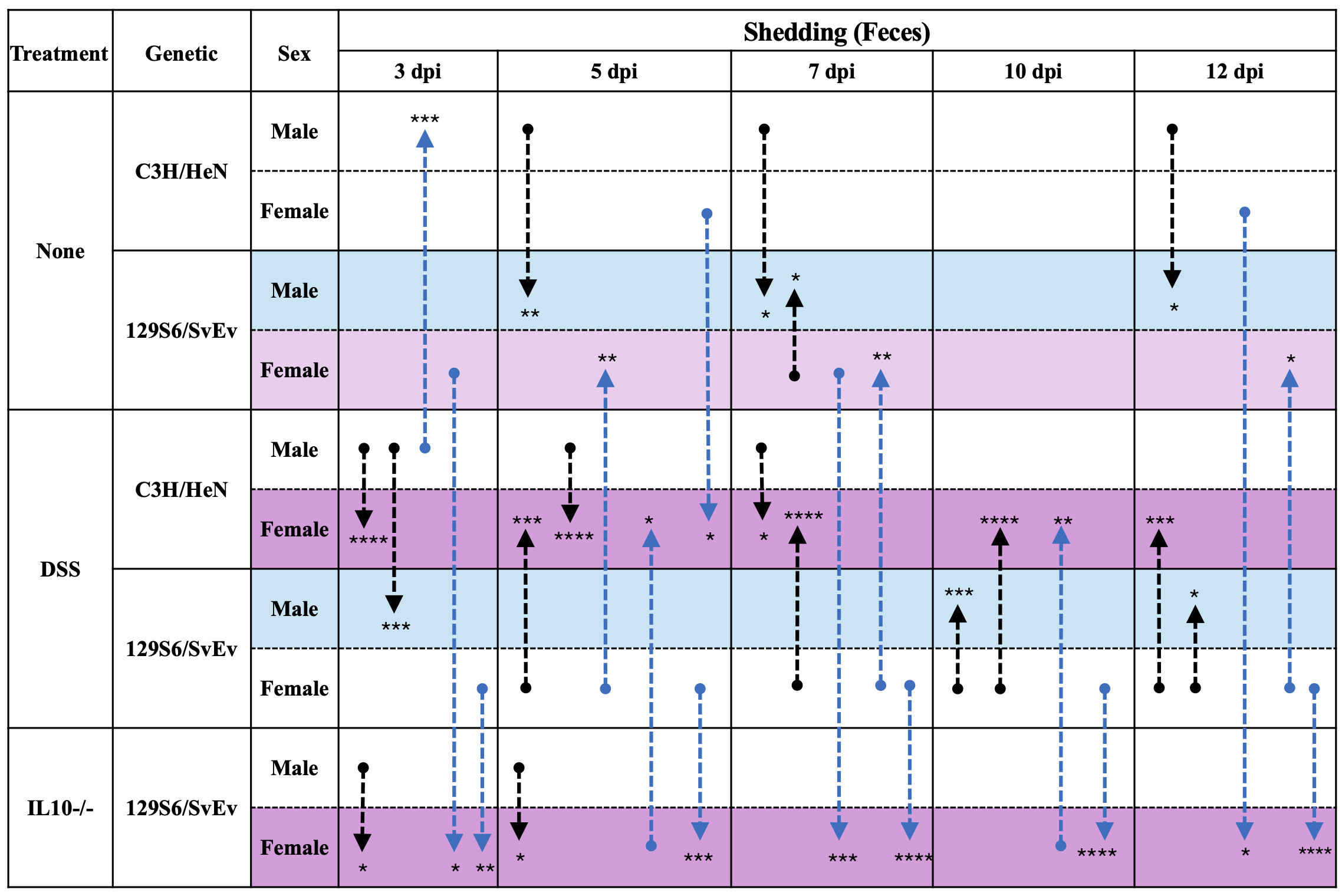

Supplement: SUPPLEMENTARY FIGURE S2 — Summary of significant comparisons in fecal shedding data. Comparisons drawn in Figures 2–5 are summarized with arrows pointing from compared group demonstrating lowest fecal shedding of SPtA at a given time point toward group demonstrating highest fecal shedding of SPtA at a given time point. IL10, interleukin 10; *, p-value < 0.05; **, p-value < 0.01; ***, p-value < 0.001; ****, p-value < 0.0001. [file Image_2.tiff]

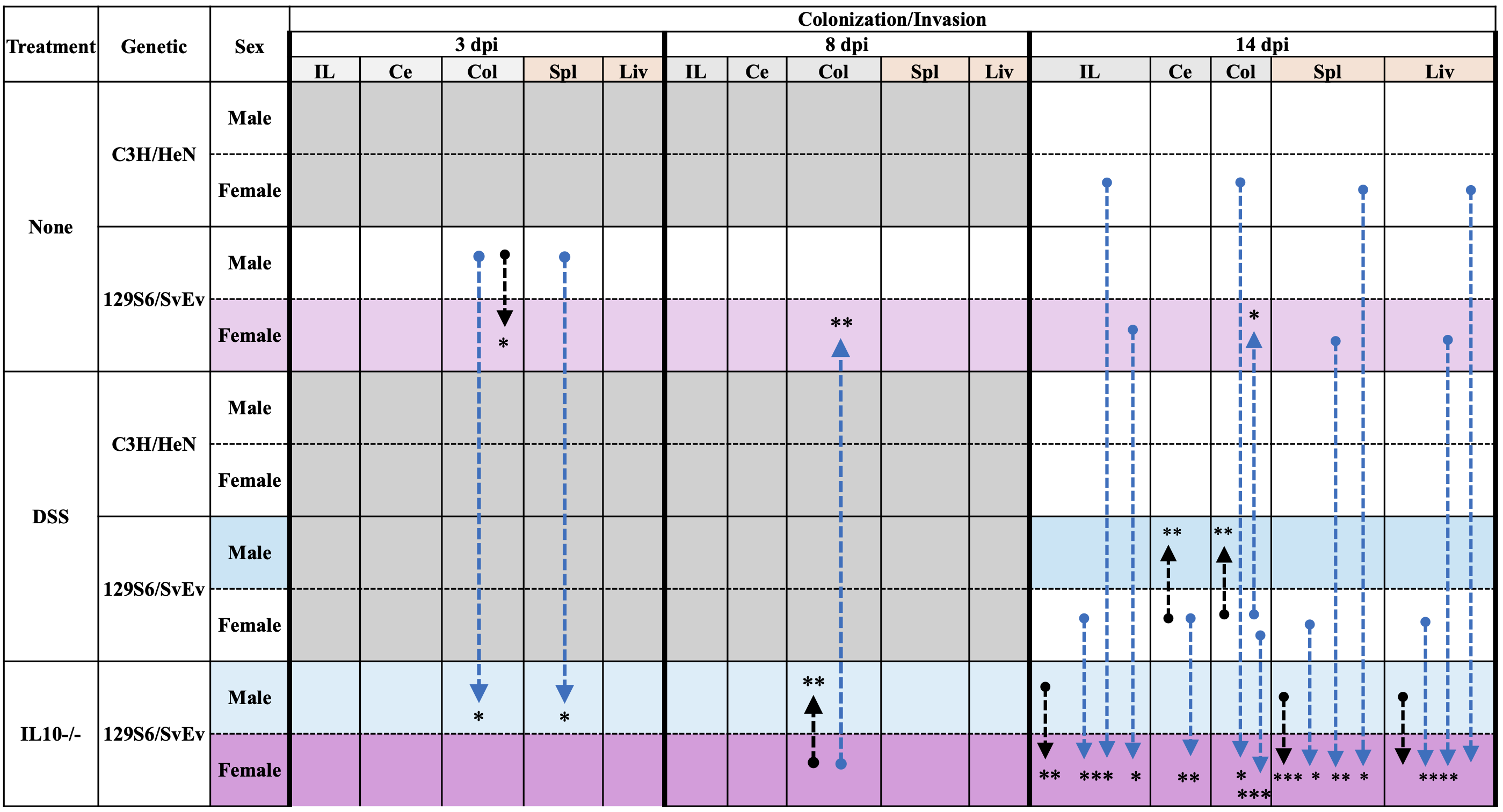

Supplement: SUPPLEMENTARY FIGURE S3 — Summary of significant comparisons in colonization data. Comparisons drawn in Figures 6–8 are summarized with arrows pointing from compared group demonstrating lowest detection of SPtA in intestinal content or a given tissue at a given time point toward group demonstrating highest detection of SPtA in intestinal content or a given tissue at a given time point. *, p-value < 0.05; **, p-value < 0.01; ***, p-value < 0.001; ****, p-value < 0.0001. [file Image_3.tiff]

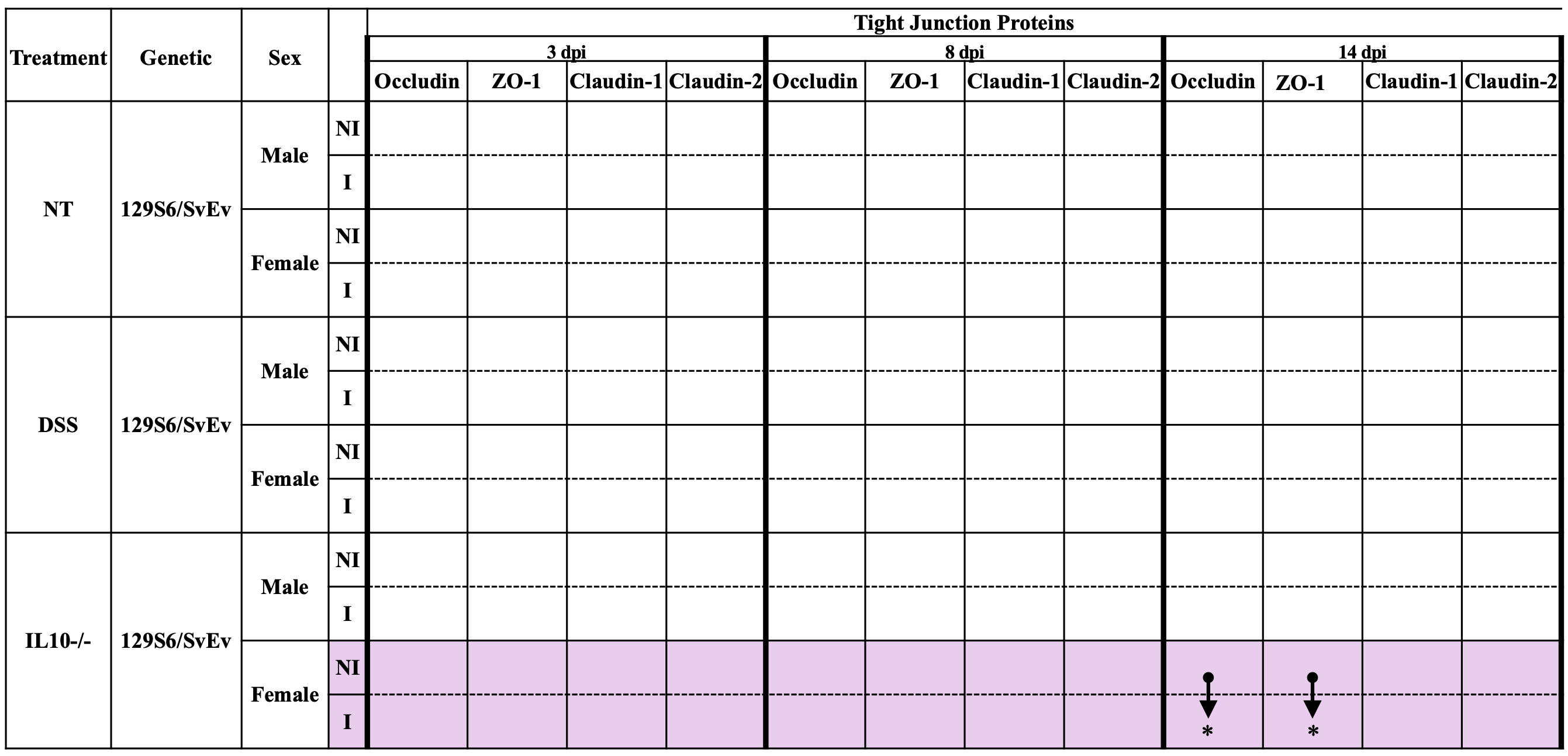

Supplement: SUPPLEMENTARY FIGURE S4 — Summary of significant comparisons in tight junction protein data. Comparisons drawn in Figure 9 are summarized with arrows pointing from compared group demonstrating lowest expression of a given tight junction protein at a given time point toward group demonstrating highest expression of a given tight junction protein at a given time point. *, p-value < 0.05; **, p-value < 0.01; ***, p-value < 0.001; ****, p-value < 0.0001. [file Image_4.tiff]

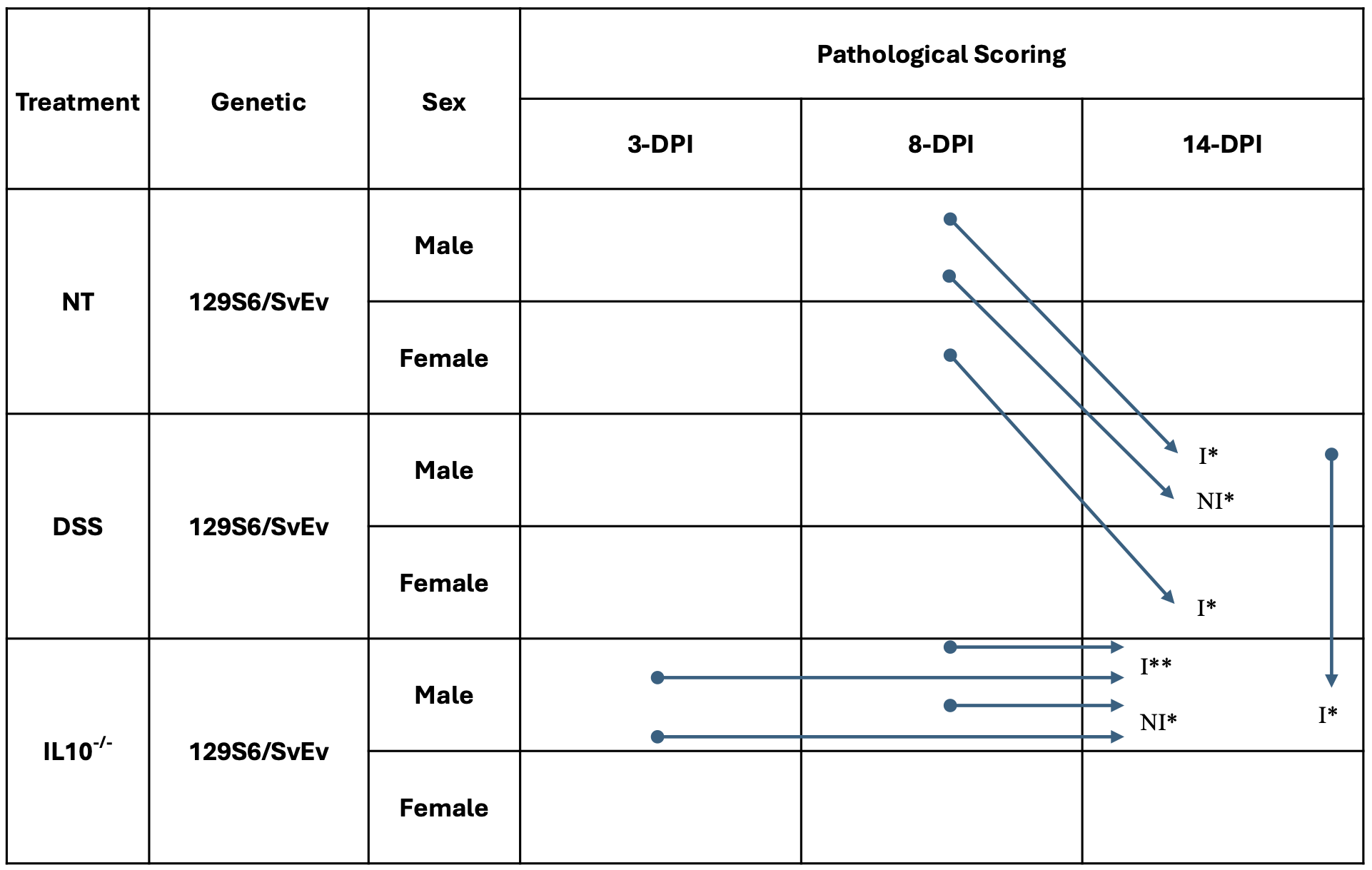

Supplement: SUPPLEMENTARY FIGURE S5 — Summary of significant comparisons in pathology data. Comparisons drawn in Figures 11, 12 are summarized with arrows pointing from compared group demonstrating lowest pathological score at a given time point toward group demonstrating highest fecal shedding at a given time point. *, p-value < 0.05; **, p-value < 0.01; ***, p-value < 0.001; ****, p-value < 0.0001. [file Image_5.tiff]

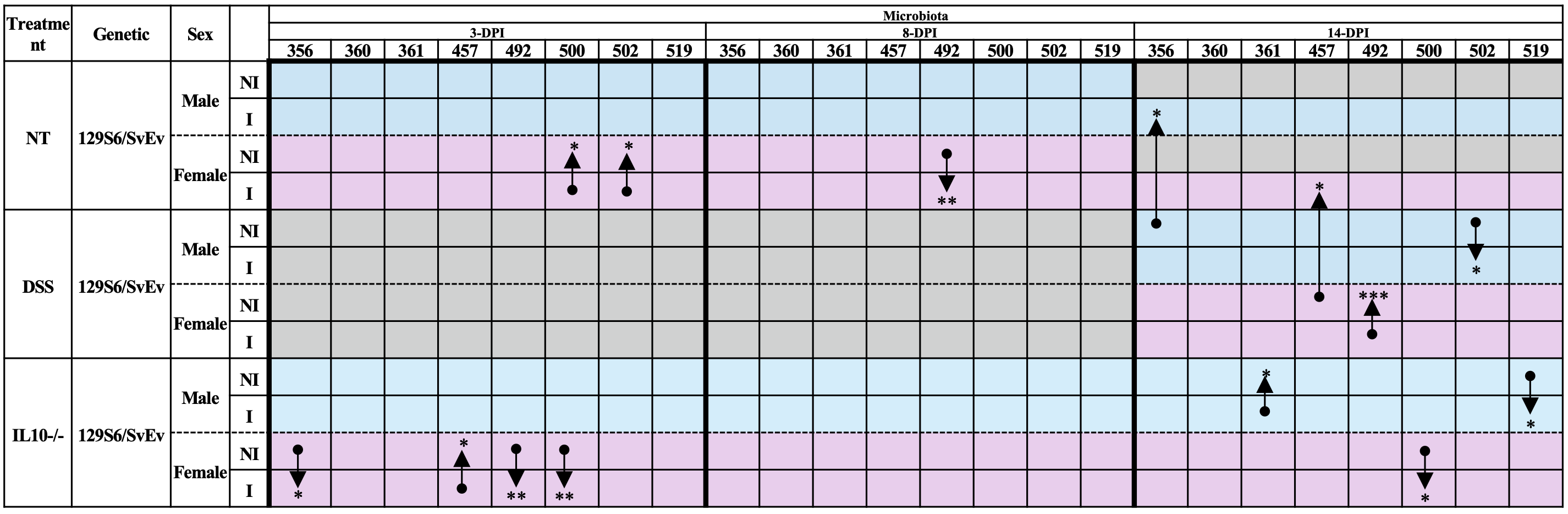

Supplement: SUPPLEMENTARY FIGURE S6 — Summary of significant comparisons in microbiota data. Comparisons drawn in Figures 13–15 are summarized with arrows pointing from compared group demonstrating lowest abundance of a given taxa at a given time point toward group abundance of a given taxa at a given time point. *, p-value < 0.05; **, p-value < 0.01; ***, p-value < 0.001; ****, p-value < 0.0001. [file Image_6.tiff]
